# Structural Regulation of Pd‐Based Nanoalloys for Advanced Electrocatalysis

**DOI:** 10.1002/smsc.202100061

**Published:** 2021-10-17

**Authors:** Menggang Li, Zhonghong Xia, Mingchuan Luo, Lin He, Lu Tao, Weiwei Yang, Yongsheng Yu, Shaojun Guo

**Affiliations:** ^1^ MIIT Key Laboratory of Critical Materials Technology for New Energy Conversion and Storage, School of Chemistry and Chemical Engineering Harbin Institute of Technology Harbin Heilongjiang 150001 China; ^2^ School of Materials Science and Engineering Peking University Beijing 100871 China

**Keywords:** dimensionality of Pd‐based nanoalloys, electrocatalysis, fuel cells, palladium

## Abstract

Palladium (Pd)‐based materials have attracted increasing attentions as a kind of novel candidate catalysts for many electrocatalytic reactions to replace classic platinum (Pt) catalysts, especially in the fuel cell‐related electrocatalysis. However, the requirement of high activity and stability toward further practical applications makes the development of Pd‐based catalysts cease to advance. Combining alloying and structure‐controlled strategies has well addressed this challenge by optimizing the adsorption/desorption behaviors toward reaction intermediates. Herein, the recent advances of rational structural designs in terms of tuning the dimensionalities of Pd‐based nanoalloys are overviewed. To further enhance the intrinsic electrocatalytic activity, several advanced strategies, including intermetallics, doping, defects, surface, and interface engineering, are presented to engineer the electronic and/or physical properties of Pd‐based electrocatalysts. Using typical electrocatalytic reactions as probes, the significance of structural regulation of Pd‐based nanocrystals on the enhanced electrocatalysis is demonstrated. Finally, several possible trends and challenges for future advanced research directions are presented. It is anticipated that the rational structural regulation can make promising Pd‐based catalysts touch the ceiling of electrocatalytic activity and stability.

## Graphical abstract

Herein, recent advances in rational structural designs of palladium (Pd)‐based nanoalloys in terms of the regulation of the dimensionalities for advanced electrocatalysis are provided. In addition, several advanced strategies, including intermetallics, doping, defects, surface, and interface engineering, are further reviewed to engineer the electronic and/or physical properties of Pd‐based electrocatalysts.

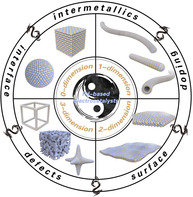

## 1 Introduction

The ever‐increasing energy consumption and environmental pollution have driven the researchers to search the economical and eco‐friendly energy generation, storage, and conversion devices.^[^ ^1^ ^]^ To develop high‐performance renewable energy technologies, such as fuel cells, water electrolyzers, and metal–air batteries, it is very necessary to design the state‐of‐the‐art electrocatalysts. For example, platinum (Pt)‐based materials are considered as the most effective electrocatalysts toward oxygen reduction reaction (ORR) and hydrogen oxidation reaction (HOR), two key electrode reactions in fuel cells.^[^ ^2^ ^]^ However, the high‐cost of Pt‐based catalysts stimulates the exploration of alternatives with satisfactory activity and lower price, such as carbon‐based composites and non‐noble transition metal‐based compounds.^[^ ^3^ ^]^ Although great success has been achieved on these earth‐abundant electrocatalysts, an obvious gap toward activity and stability still exists. Palladium (Pd), located in the same group to Pt, shares the similar physicochemical properties with Pt and the higher oxidation potentials compared with the other transition metals.^[^ ^4^ ^]^ Therefore, Pd and Pd‐based materials gain increasing attentions as potential candidates for replacing Pt toward electrocatalysis. Although the ORR activity of Pd‐based catalysts is lower than that of Pt catalysts in acid condition, it can show a comparable activity with that of Pt in alkaline solution.^[^ ^5^ ^]^ This is because that the Pd possesses stronger binding energy of oxygen containing species than that of Pt in acid solution, and shows the decreased anion poisoning effect in alkaline solution. More importantly, the historical cost of Pd metals is only 1/2–1/3 of Pt metals, making Pd‐based catalysts the most attractive candidates for replacing Pt.^[^ ^5^ ^]^ Therefore, developing novel Pd‐based materials as high‐performance electrocatalysts is of significance for next‐generation energy conversion devices.

Despite the great achievements, the developed Pd‐based materials can hardly meet high demand of desired electrocatalysts with excellent activity and stability, especially in practical applications. Therefore, it is very necessary to reduce the Pd utilization while maintaining the high catalytic activity, leaving an important task for the design and regulation of novel Pd‐based nanostructures. In this case, two aspects about available active sites should be considered, including the numbers of electrocatalytically active sites and inherent activity of each active sites.^[^ ^6^ ^]^ By tuning the surface coordination environments of the catalysts, various strategies have been proposed to optimize the adsorption/desorption processes toward the reaction intermediates.^[^ ^7^ ^]^ For instance, the faceted effect has an important influence on the catalytic activity in acidic electrolyte with the order of Pd (100) > Pd (111) > Pd (110), in opposition to that of Pt catalysts.^5^, ^8^ The facet‐dependent mechanism on electrocatalysis is mainly originated from the distinction of surface atom coordination on different single‐crystalline facets. Compared with these low‐indexed facets, high‐indexed nanocrystals display higher catalytic activity due to the adjacent low‐coordinated atoms located at steps, kinks, and edges.^[^ ^9^ ^]^ Moreover, the poor durability during long‐term tests of Pd‐based electrocatalysts is another noteworthy issue. In particular, the active surface of Pd electrodes will be poisoned by the CO‐related intermediates released from the process of alcohol oxidation electrocatalysis, leading to a sharp decline of activity.^[^ ^10^ ^]^ The aforementioned issues inspire more advanced strategies to achieve Pd‐based catalysts with high activity and durability, as well as the reduced cost.

It has been confirmed that the adsorption/desorption behaviors, the key for determining the catalytic activity, are fundamentally governed by the surface structures of catalysts.^[^ ^11^ ^]^ Consequently, the catalytic performance of a desired catalyst can be maximized by engineering the geometric and electronic structure (such as *d*‐band center of noble metal‐based electrocatalysts). In the past few decades, a large number of strategies for structural regulation of catalysts have been reported, culminating in the advent of the regulation at atomic level.^[^ ^12^ ^]^ Alloying is the most efficient method for optimizing the adsorption/desorption behaviors toward reaction intermediates. Combining alloying and morphology controlling, the inherent activity of active sites and the numbers of electrocatalytically active sites are synergistically boosted. More importantly, several more advanced strategies, involving intermetallics, doping, defects, surface, and interface engineering, can further engineer the electronic properties of Pd‐based nanoalloys, thus expanding the generated active sites and improving the physicochemical properties at nanoscale.^[^ ^13^ ^]^

In this review, we focus on the recent developments in controlling morphologies of Pd‐based nanomaterials, covering 0D, 1D, 2D, and 3D structures. Furthermore, we also highlight how the structural regulation can boost the electrocatalytic performance of Pd‐based electrocatalysts. By classifying these structural regulation strategies, we show the vital function of engineered geometric and electronic properties of Pd‐based catalysts. We also discuss the relationship between these advanced strategies and the enhanced electrocatalytic performance. Finally, we make a short conclusion as well as a perspective of future key challenges in this hot research direction.

## 2 Pd‐Based Nanoalloys for Electrocatalysis

**Scheme 1:**
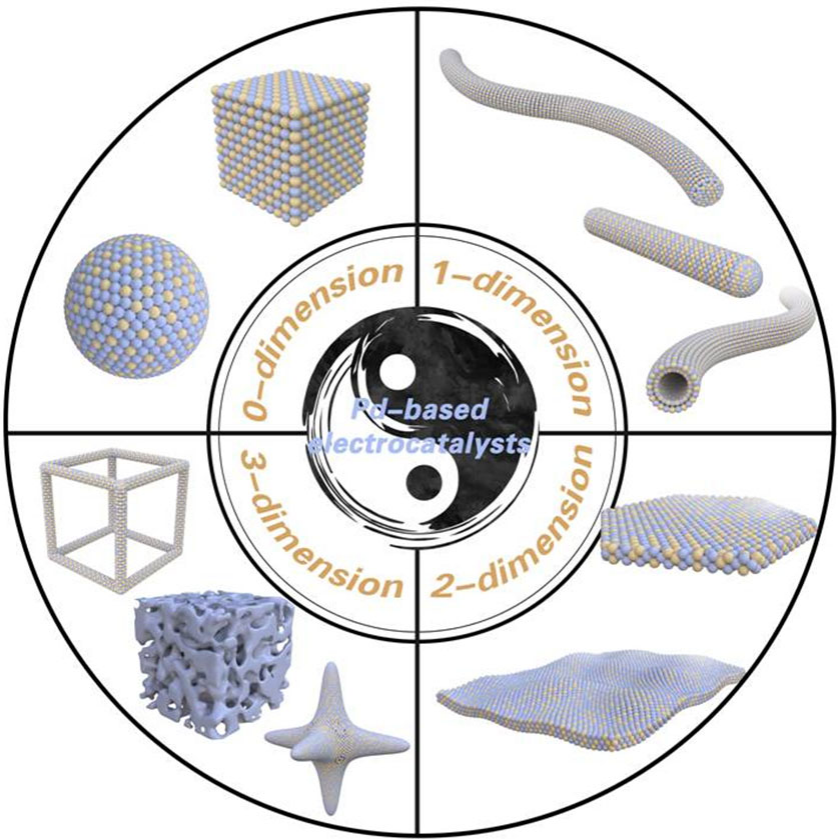
Scheme of morphology‐controlled Pd‐based nanoalloys for efficient electrocatalysis at different dimensionality.

To achieve high‐performance Pd‐based electrocatalysts, an efficient method is to alloy Pd with other elements, such as Fe, Cu, Pb, Ag, Au, Mo, and W.^[^ ^14^ ^]^ Through incorporating additional elements into the host Pd nanomaterials, the electronic structure of the surface or near‐surface atoms can be regulated. Therefore, the generated ligand/strain effect results in the optimization of adsorption/desorption ability toward the reaction intermediates. This is the key for the enhanced electrocatalyic activity on the alloyed catalysts. In this section, considering the dimensionality, we selectively review the morphology‐controlled synthesis of Pd‐based nanoalloys for electrocatalysis (**Scheme** **1**). The emphases for our discussion are the synthetic methods of various Pd‐based nanoalloys and the corresponding structural properties beneficial for the enhanced electrocatalysis.

### 2.1 0D Nanostructures

As is known, the truncated octahedron is an equilibrium shape of a single crystal with the face‐centered cubic (fcc) structure, such as Pd crystals.^[^ ^15^ ^]^ Therefore, the 0D nanoalloys are relatively easy to be synthesized and stabilized. This is also the reason for that 0D nanostructures are the most common existing forms of Pd‐based electrocatalysts, such as the commercial Pd/C catalyst is the carbon‐supported 3 nm Pd nanoparticles. However, pure Pd catalysts have a very strong binding toward electrocatalytic reaction intermediates, especially for Pd(111) surface. In terms of ORR, it is better to lower the position of the *d*‐band center of Pd catalysts relative to the Fermi level, so that that the complex reaction intermediates, including *O, *OH, and *OOH, can be desorbed easily. This can be addressed by obtaining Pd‐based alloyed nanostructures to shift Pd catalysts to the optimized binding energy. By alloying Pd with Co elements, the Pd—Pd interatomic distance was optimized to absorb the oxygen species, resulting in the enhanced ORR activity on the optimized Pd_2_Co bimetallic nanoparticles.^[^ ^16^ ^]^ With respect to Pd—Fe alloyed nanoparticles, the highest ORR activity was obtained on the Pd_3_Fe nanoalloys.^[^ ^17^ ^]^ Zhou et al. further demonstrated that the ORR kinetics of a segregated Pd (111) layer formed on the surface of high‐temperature‐annealed Pd_3_Fe (111) single‐crystal alloys was faster than that of pristine Pd (111).^[^ ^18^ ^]^ The modified electronic structure of Pd surface by the underlying Fe atoms weakened the Pd—OH bond, thus achieving the enhanced catalytic activity. This result is in agreement with the theory that the position of the *d*‐band center directly related to the adsorption energy.^[^ ^19^ ^]^ The well consistency of experiments and theories confirmed the advantages of alloyed Pd‐based nanomaterials for describing trends in enhancing electrocatalytic activity.

Most of the Pd‐based alloys show the typical fcc crystal structure with the (111) surface as the most important facets. However, there are also two important facets, (100) and (110) facets, exposed on the surface of 0D Pd‐based nanostructures. As mentioned earlier, the faceted effect has an important influence on the catalytic activity. To demonstrate the facet‐controlled synthesis of Pd‐based nanocrystals via thermodynamically or kinetically controlled reactions, several key factors must be considered to dominate the nucleation.^[^ ^20^ ^]^ For example, the amounts of crystal seeds for inducing the formation of polyhedron, the reaction temperature, and the presence of halide ions are beneficial for capping the specific facets.^[^ ^21^ ^]^ In particular, the halides can selectively block the surface by changing the surface energy of different exposed facets. Therefore, the Pd‐based nanocrystals with different exposed facets can be obtained. A typical example is that the presence of Br^−^ can lead to the formation of Pd nanocubes enclosed by the (100) facets, while only Pd cuboctahedron enclosed by the both (100) and (111) facets was formed under the absence of Br^−^.^[^ ^22^ ^]^ As the conceptual demonstration, the halide ions also played an important role in the formation of PdPt bimetallic nanocubes.^[^ ^23^ ^]^ As‐obtained PdPt nanocubes were evaluated as high‐performance electrocatalysts for the ORR with the activity is 4.0 times higher than that of the commercial Pt/C. Furthermore, compared with PdCu nanoparticles, the PdCu nanocubes showed higher ORR activity due to the exposure of (100) facets, in well agreement with the previous reports.^[^ ^24^ ^]^

### 2.2 1D Nanostructures

Compared with the traditional 0D nanostructures, 1D nanomaterials show many unique properties, such as the enhanced electron and mass transport, as well as the improved chemical stability.^[^ ^25^ ^]^ In addition, anisotropic 1D nanocrystals also provide multiple anchoring points for the carbon supports, thus are anticipated to achieve high electrocatalytic activity and stability. However, it is difficult and challenging to obtain anisotropic nanocatalysts using the wet‐chemical methods due to the intrinsically thermodynamic instability of anisotropic structures and the isotropic growth behaviors of metals, especially those with high aspect ratio.^[^ ^26^ ^]^ To this end, various Pd‐based 1D nanostructures, including nanowires, nanorods, nanotubes, and so on, were constructed by optimizing the synthetic methods and their electrocatalytic behaviors were further studied.

**Figure 1:**
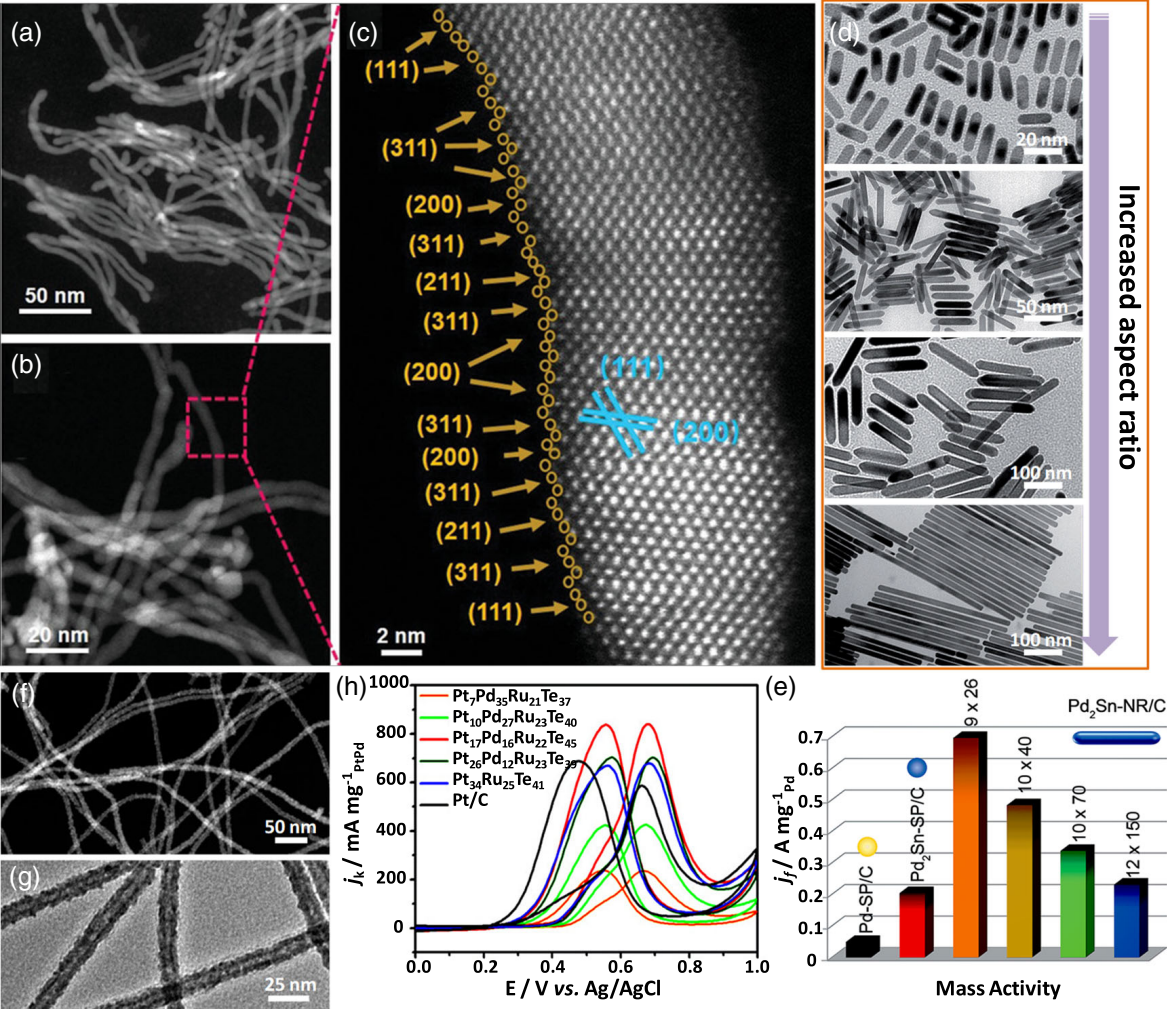
The 1D Pd‐based nanostructures for efficient electrocatalysis. a) Low‐ and b) high‐magnification high‐angle annular dark‐field scanning transmission electron microscopy (HAADF‐STEM) images of Pd_4_Ag nanowires and c) aberration‐corrected HAADF‐STEM image of a single Pd_4_Ag nanowire from the enclosed region in (b). a–c) Reproduced with permission.^[^ ^28^ ^]^ Copyright 2021, Wiley‐VCH. d) Transmission electron microscopy (TEM) images of Pd_2_Sn nanorods with different aspect ratio (from top to bottom are 9/26, 10/70, 12/150, and 20/460, respectively) and e) EOR mass activities of different Pd_2_Sn nanorods, Pd_2_Sn nanoparticles, and pure Pd nanoparticles. d,e) Reproduced with permission.^[^ ^32^ ^]^ Copyright 2021, The Royal Society of Chemistry. f) HAADF‐STEM image of Pt_17_Pd_16_Ru_22_Te_45_ nanotubes, g) TEM image of Pt_26_Pd_12_Ru_23_Te_39_ nanotubes, and h) MOR curves of different PtPdRuTe nanotubes, PtRuTe nanotubes, and commercial Pt/C catalysts in 0.5 m H_2_SO_4_ + 1.0 m CH_3_OH at a scan rate of 50 mV s^−1^. f–h) Reproduced with permission.^[^ ^34^ ^]^ Copyright 2017, American Chemical Society.

As the most common 1D nanostructures, Pd‐based nanowire catalysts attracted great attention in recent years. The hard template‐assisted synthetic technology is an exciting method to obtain nanowires by tuning the basic morphology of the corresponding templates. By coupling the template‐assisted approach and chemical deposition, the bimetallic Pd—Au and Pd—Pt nanowire arrays with various chemical compositions were prepared.^[^ ^27^ ^]^ The average diameter and length of as‐obtained nanowire arrays were ≈50 nm and ≈6 μm, respectively. When used as ORR electrocatalysts, both the Pd—Au and Pd—Pt nanowire arrays exhibited better electrocatalytic performance compared with pure Pt catalysts. In addition, in terms of soft‐template methods, Pd—Ag alloyed nanowires with large aspect ratio and roughened surfaces were synthesized via reducing AgNO_3_ and H_2_PdCl_4_ precursors under the presence of dihexadecyldimethylammonium chloride (DHDAC) (**Figure** **1**a–c).^[^ ^28^ ^]^ Alloyed Pd—Ag nanowires induced the charge transfer from Ag to Pd, leading to a downshifted *d*‐band center of Pd catalysts and the diluted PdH_*x*_ catalytic sites. Therefore, the optimized Pd_4_Ag nanowires initiated CO_2_ reduction reaction (CO_2_RR) at equilibrated overpotential and achieved high selectivity toward formate, as well as excellent stability tested even at potentials <−0.2 V. On the contrary, researches are in the pursuit of ultrathin nanostructures, with the purpose of obtaining larger exposure of surface active atoms. Generally, the abundant surface atoms are beneficial for boosting the electrochemical specific surface areas (ECSAs), thereby achieving the enhanced electrocatalytic activity. To achieve highly efficient electrocatalysis toward ethanol oxidation reaction (EOR), ultrathin single‐crystal PdAg alloyed nanowires with the features of ultralong and high‐density undercoordinated surface atoms were successfully synthesized.^[^ ^29^ ^]^ Due to the synergic effect of electronic and bifunctional properties of PdAg nanowires, the adsorption and diffusion of intermediates were kinetically activated. In addition, alloying oxophilic Ag metals optimized the electronic structure of Pd, which thermodynamically weakened the adsorption of CH_3_CO_ads_ and OH_ads_, resulting in an accelerated electrocatalysis. Therefore, a highest mass activity toward EOR of 2.84 A mg_Pd_^−1^ was achieved on the optimized Pd_2_Ag_1_ nanowires, better than those of polycrystalline PdAg nanowires, PdAg nanoparticles, and pure Pd nanowires.

Different from nanowires, nanorods often have the smaller aspect ratios (usually less than 10), enabling the production of catalysts with robust structural stability and relatively large exposed active sites.^[^ ^30^ ^]^ Typically, Pd nanorods can be synthesized via the electrochemical deposition, in which PdCl_2_ as the metal precursors.^[8c]^ By tuning the concentration of PdCl_2_, the morphology of products was transformed from nanoparticles to nanorods. The well‐constructed Pd nanorods showed Pt‐like ORR activity, much higher than that of Pd nanoparticles. Using the Pd nanowires as crystal seeds, PtPd porous nanorods were synthesized by the bromide‐induced galvanic replacement reaction in the K_2_PtCl_6_ aqueous solution.^[^ ^31^ ^]^ The porous structures provided the large ECSA, thus the alloyed PtPd nanorods can be served as highly active and stable ORR electrocatalysts. The enlarged surface area, the enhanced catalytic activity, and the improved durability enabled such PtPd porous nanorods to be potential cathode ORR electrocatalysts for fuel cells. Furthermore, the Pd_2_Sn nanorods with different aspect ratio were synthesized by changing the feeding amount of methylamine hydrochloride (MAHC) (Figure 1d).^[^ ^32^ ^]^ This work provided a novel direct wet‐chemical approach to design Pd‐based nanorods. The exposed (100) and (001) facets of orthorhombic Pd_2_Sn nanorods showed more favorable adsorption toward ethanol and OH^−^ intermediates according to the density functional theory (DFT) calculations. Therefore, the excellent EOR activity, 3.0 and 10.0 times higher than those of Pd_2_Sn and Pd nanospheres, can be achieved on the optimized Pd_2_Sn nanorod catalysts (Figure 1e).

Combining 1D and hollow structures, nanotubes can be obtained to restrain mechanical degradation and prevent volume expansion. These advantages are enough to make the nanotubes be promising unsupported electrocatalysts due to the avoiding corrosion of the carbon supports.^[25a]^ To synthesize Pd‐based nanotubes, the galvanic replacement and the Kirkendall effect are the most classic methods. By means of the galvanic displacement of Cu nanowires, Pd nanotubes were synthesized and showed the enlarged surface area for highly active HOR electrocatalysis, 20.0 times greater than that of Pd nanoparticles.^[^ ^33^ ^]^ Alloying is of significance for boosting electrocatalytic activity. The well‐defined quaternary PtPdRuTe nanotubes with different compositions were further synthesized by the similar galvanic replacement reaction using the ultrathin Te nanowires as the sacrificial templates (Figure 1f,g).^[^ ^34^ ^]^ The synergy between several elements endowed the alloyed PtPdRuTe nanotubes the enhanced catalytic activity and durability toward methanol oxidation reaction (MOR) (Figure 1h). Specifically, the residual Te atoms served as skeleton minimize the utilization of other noble metals. The key active sites toward MOR were the formed Pt—CO bonds due to the presence of Pt atoms, while oxyphilic Ru atoms facilitated the adsorption of oxygen‐contained species. More interestingly, the introduction of Pd atoms with higher reduction potential further modified the electronic properties of Pt atoms, thus stabilizing the surface of nanotubes. The localized surface plasmon resonance excitation strategy was further used for regulating the MOR electrocatalysis of alloyed Pd—Ag nanotubes.^[^ ^35^ ^]^ The enhanced activity under excitation was attributed to the hot holes (h^+^) generated in Pd—Ag nanotubes by the excitation of plasma.

### 2.3 2D Nanostructures

**Figure 2:**
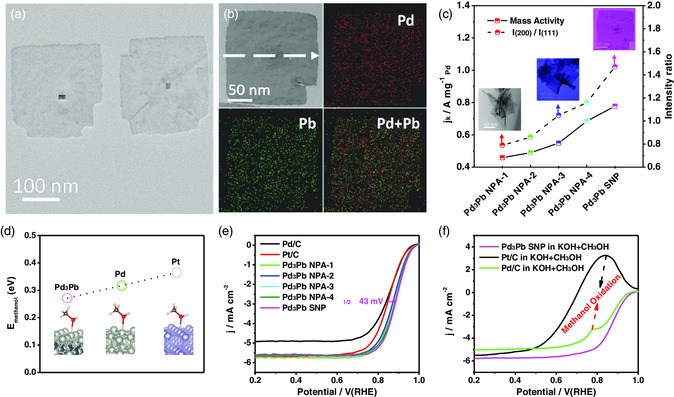
The 2D Pd‐based nanoplates for efficient electrocatalysis. a) TEM image and b) STEM energy‐dispersive X‐ray spectroscopy (STEM‐EDS) elemental mapping of Pd_3_Pb square nanoplates. c) The power X‐ray diffraction (PXRD) intensity ratio of *I*_(100)_/*I*_(111)_ and *I*_(200)_/*I*_(111)_ of different Pd_3_Pb nanoplates (NPA and SNP represent nanoplate assemblies and square nanoplates, respectively) (the insets are the corresponding TEM images). d) The adsorption energy of methanol (*E*_methanol_) of Pd_3_Pb (100), Pd (111), and Pt (111) surfaces. e) ORR polarization curves of different Pd_3_Pb nanoplates, commercial Pd/C and Pt/C catalysts in O_2_‐saturated 0.1 m KOH with a scan rate of 10 mV s^−1^. f) ORR polarization curves of Pd_3_Pb square nanoplates, commercial Pd/C and Pt/C in O_2_‐saturated 0.1 m KOH + 0.5 m CH_3_OH with a scan rate of 10 mV s^−1^. a–f) Reproduced with permission.^[^ ^37^ ^]^ Copyright 2018, Wiley‐VCH.

It is well known that 2D nanostructures possess abundant low‐coordinated atoms at the perimeters, as well as high surface area and exposed active sites. Therefore, 2D Pd‐based nanocrystals attracted increasing attentions in the electrocatalysis, such as nanoplates and nanosheets. For instance, Pd nanoplate arrays were directly grown onto the Au substrate via an electrochemical method without the templates.^[^ ^36^ ^]^ The formation of Pd nanoplate arrays was highly dependent on the employment of applied potentials and the feeding amount of surfactant (cetyltrimethylammonium bromide [CTAB]). Effected by the uniform electric field in this synthetic system, the products would be gradually transformed from nanoparticles to nanoplates. Due to the highly rough surface, the MOR activity of Pd nanoplate arrays was several times higher than that of flat Pd film catalysts. In terms of alloyed Pd‐based nanoplates, Pd_3_Pb square nanoplates with an edge length of ≈200 nm and the thickness of ≈5.2 nm were synthesized via a one‐pot wet‐chemical method (**Figure** **2**a,b).^[^ ^37^ ^]^ With increasing the feeding amount of Pb precursors, the morphology of Pd_3_Pb changed from nanoplate assemblies to square nanoplates with increased (100) facet orientation (Figure 2c). Electrocatalytic tests indicated that the ORR performance of different Pd_3_Pb nanoplates was dependent by the crystalline orientation with the (100)‐facet‐dominated square nanoplates being the most active. More importantly, the 3*d* states of Pd_3_Pb (100) surface were closer to the Fermi level than those of Pd (111) and Pt (111) surfaces, very helpful for the improved methanol tolerance properties (Figure 2d). Therefore, the well‐designed Pd_3_Pb square nanoplates with a (100) preferred orientation exhibited the highest ORR activity of 0.78 A mg^−1^ at 0.9 V versus reversible hydrogen electrode (RHE), as well as the excellent methanol tolerance (Figure 2e,f).

**Figure 3:**
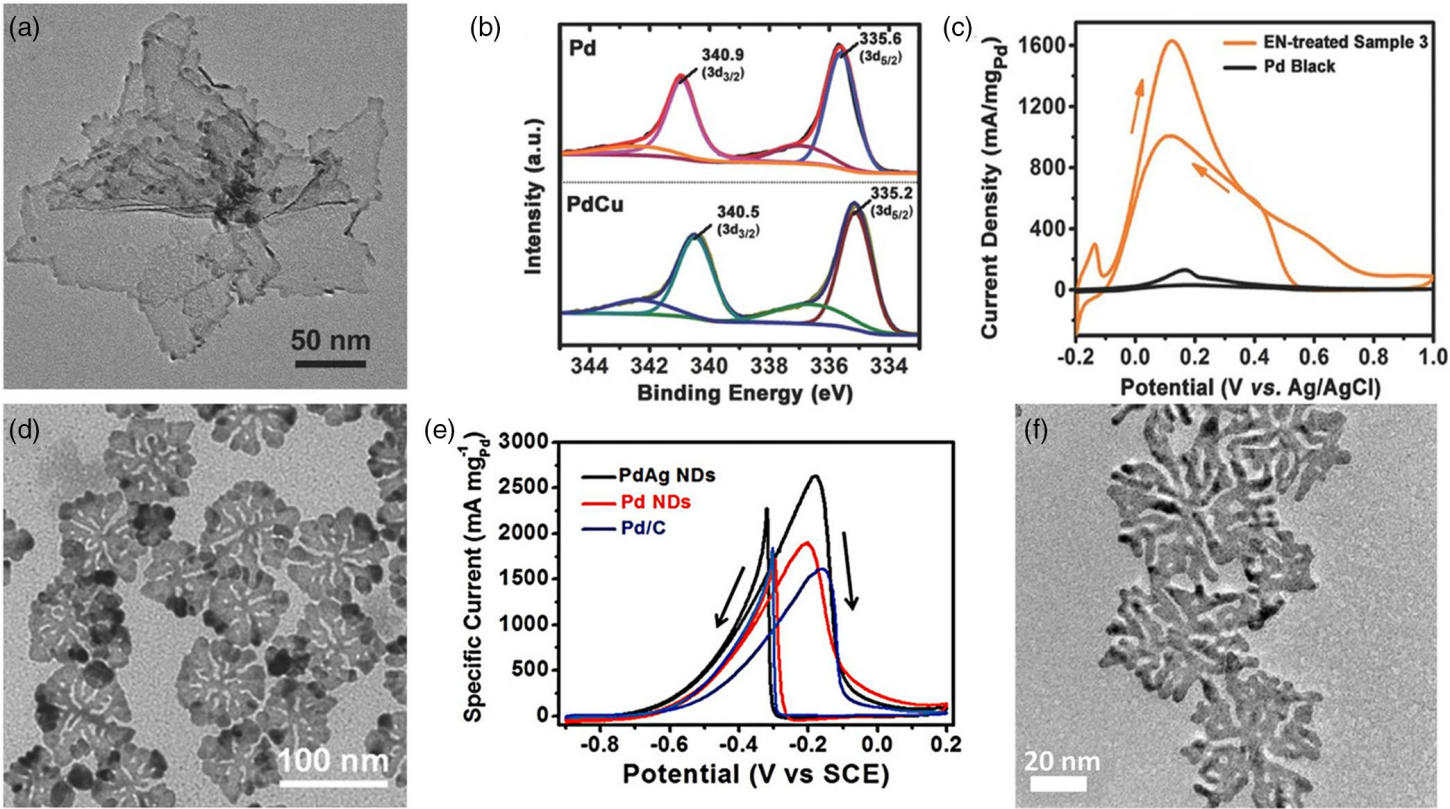
The 2D Pd‐based nanosheets and nanodendrites for efficient electrocatalysis. a) TEM image of PdCu nanosheets, b) Pd 3*d* XPS spectra of Pd and PdCu nanosheets and c) FAOR curves of PdCu nanosheets and commercial Pd black catalysts in 0.5 m H_2_SO_4_ + 0.25 m HCOOH with a scan rate of 50 mV s^−1^ (EN‐treated sample 3 represents PdCu nanosheets treated by ethylenediamine). a–c) Reproduced with permission.^[^ ^38^ ^]^ Copyright 2017, Wiley‐VCH. d) TEM image of 2D PdAg nanodendrites and e) EOR curves of 2D PdAg, Pd nanodendrites, and commercial Pd/C catalysts in 1.0 m KOH + 1.0 m ethanol at a scan rate of 20 mV s^−1^ (NDs represents nanodendrites). d,e) Reproduced with permission.^[45a]^ Copyright 2018, Wiley‐VCH. f) TEM image of 2D PdCu nanodendrites. Reproduced with permission.^[^ ^46^ ^]^ Copyright 2021, American Chemical Society.

Different from relatively thick nanoplates, nanosheets exhibit smaller thickness, resulting in a higher specific surface area per mass. Inspired by the unique properties of nanosheets, the uniform PdCu alloyed nanosheets were synthesized via controlling the atomic ratio of Pd/Cu (**Figure** **3**a).^[^ ^38^ ^]^ The charges in PdCu nanosheets were transferred from Cu atoms to Pd atoms, leading to a distinct change in electronic properties (Figure 3b). Compared with the pristine Pd nanosheets, alloyed PdCu nanosheets showed higher formic acid oxidation reaction (FAOR) mass activities of 1493.5 ± 38.0 mA mg_metal_^−1^ and 1655.7 ± 74.6 mA mg_Pd_^−1^ (Figure 3c). Furthermore, by introducing additional 3*d* transition metals (Fe, Co, and Ni), the electronic structure of alloyed PdRu nanosheets was modulated to ensure the continuous electron‐supply sites of catalysts.^[^ ^39^ ^]^ In the enhanced hydrogen evolution reaction (HER) catalytic system, stabilized Pd centers were beneficial for the initial dissociation of H_2_O molecules. In addition, the enriched electrons were effectively transferred to the adsorbed species. The optimized trimetallic Ru_38_Pd_34_Ni_28_ nanosheets showed low overpotential of 20 mV at a current density of 10 mA cm^−2^ and a high mass activity of 6.15 A mg^−1^ at 0.07 V versus RHE, as well as the excellent stability. The similar behavior can also be found in the single‐atom‐layer Pd‐based alloys, where the formic acid oxidation reaction (FAOR) mass activity of single‐atom‐layer PdCo alloy was eight times higher than that of Pd catalysts.^[^ ^40^ ^]^

One of the factors affecting electrocatalytic activity is the numbers of catalytically active sites. Creating the ultrathin systems represents a potential trend to increase the ECSAs, resulting in a more attractive electrocatalytic activity.^[^ ^41^ ^]^ On this basis, PdMo bimetallene with highly curved and subnanometer‐thick features was synthesized via a one‐pot wet‐chemical method.^[14e]^ The thickness of as‐synthesized PdMo bimetallene was only 0.88 nm. X‐ray photoelectron spectroscopy (XPS) results indicated that the Pd 3*d* spectra of PdMo bimetallene shifted positively relative to that of Pd metallene, implying the downshift of *d*‐band center in bimetallene. Due to the alloying effect and surface strain effect caused by the curve geometry, as well as the quantum size effect, the PdMo bimetallene exhibited very high ORR mass activity of 16.37 ± 0.60 A mg^−1^_Pd_, 77.9 and 327.4 times higher than those of commercial Pt/C and Pd/C. Afterward, various multimetallene came into being, and have shown breakthroughs in the various electrocatalytic reactions, such as PdZnCr, RhPdH, and PdIr metallene.^[^ ^42^ ^]^ In addition to reducing the thickness of nanosheets with lower atomic coordination characteristics, creating porous structures is another method to increase electrocatalytically active sites. This is because that the catalytic sites can be exposed at both interior and exterior surfaces of pore positions. In this case, ultrathin porous Pd metallene with tunable electronic structure showed a superior ORR activity of 0.892 A mg^−1^_Pd_.^[^ ^43^ ^]^ Note that the porous Pd metallene possessed larger ECSA compared with the pure Pd nanosheets due to the ultrathin and porous features. More interestingly, various Pd‐based ultrathin porous nanosheets (including Pd_3_Pb, Pd_3_Sn, and PdCd) were synthesized by the template‐directed strategy.^[^ ^44^ ^]^ The essence of the formation of Pd‐based porous nanosheets was the use of preformed Pd nanosheet seeds, which can serve as the templates to deposit/diffuse the foreign atoms. Among various Pd‐based porous nanosheets, the Pd_3_Pb showed the most excellent ORR mass activity and specific activity, around 6.8 and 9.8 times higher than those of the commercial Pt/C. The DFT calculations revealed that the enhanced ORR performance was mainly originated from the optimized electronic structure caused by the interatomic interaction between different metal atoms. The porous ultrathin nanosheet structures provide a powerful catalyst model to present great potential for practical applications in electrocatalysis.

In addition to traditional nanoplates and nanosheets, 2D metallic nanodendrites are a rare class of 2D alloyed materials with highly anisotropic structures.^[^ ^45^ ^]^ These unique 2D dendritic nanostructures possess abundant active sites and undercoordinated sites, beneficial for the construction of defects. Therefore, this new class of 2D Pd‐based nanomaterials may provide valuable references for electrocatalytic applications. However, the formation of 2D metallic nanodendrites is very challenging because it is difficult to ensure the kinetic separation of thermodynamically unstable 2D dendrites while suppressing crystal growth along the normal direction. This is highly dominated by the in‐plane growth rate and the slowly in‐plane growth rate can lead to the formation of traditional nanoplates or nanosheets. Therefore, it is necessary to choose appropriate structural directing agents to accelerate the growth rate of the plane. To address this challenge, 2D PdAg alloy nanodendrites were synthesized by introducing octadecyltrimethylammonium chloride (OTAC) as the structure directing agent in aqueous solution.^[45a]^ As‐obtained 2D PdAg alloy nanodendrites feature small thickness of 5–7 nm and random in‐plane branching (Figure 3d). The key for the formation of such structure is the use of the cationic surfactant with a long alkyl chain. Due to the synergistic between the unique dendritic structures and alloyed components, the PdAg nanodendrites showed the highest EOR mass activity of 2600 mA mg_Pd_^−1^ and the excellent operation stability (Figure 3e). Furthermore, the PdCu with similar 2D dendritic structures can be synthesized using the similar methods (Figure 3f).^[^ ^46^ ^]^ The well‐designed 2D PdCu alloyed nanodendrites exhibited remarkable stability and selectivity toward electrocatalytizing CO_2_ reduction to formate at a potential of −0.4 V.

### 2.4 3D Nanostructures

The complicated structures of 3D nanostructures tend to generate unique physicochemical properties. However, due to the relatively complex structures, the rational design, especially the precisely morphology‐controlled synthesis, of 3D Pd‐based nanostructures is still difficult. The common 3D nanostructures mainly include porous structures, nanodendrites, nanocages/nanoframes, nanoflowers, nanosperes, aerogels, and so on.^[^ ^47^ ^]^ Dealloying is the selective removal of alloying metals with a lower reduction potential from Pd‐based alloys via an electrochemical or a chemical approach, which tends to create nanoporous structures.^8^, ^48^ Using the Pd_20_Ni_80_ bimetallic alloy as precursors, nanoporous PdNi bimetallic catalysts were obtained by one‐step electrochemical dealloying in an acid solution for the enhanced electrocatalytic activities.^[^ ^49^ ^]^ The electronic structure of surface Pd atomic layer was modified by the underneath Ni atoms by the alloying effect and surface strain effect. The transferred charges can increase electrocatalytically active sites for the adsorption of intermediates. As a result, the porous PdNi displayed strong potential in the enhanced ORR and FAOR electrocatalysis.

**Figure 4:**
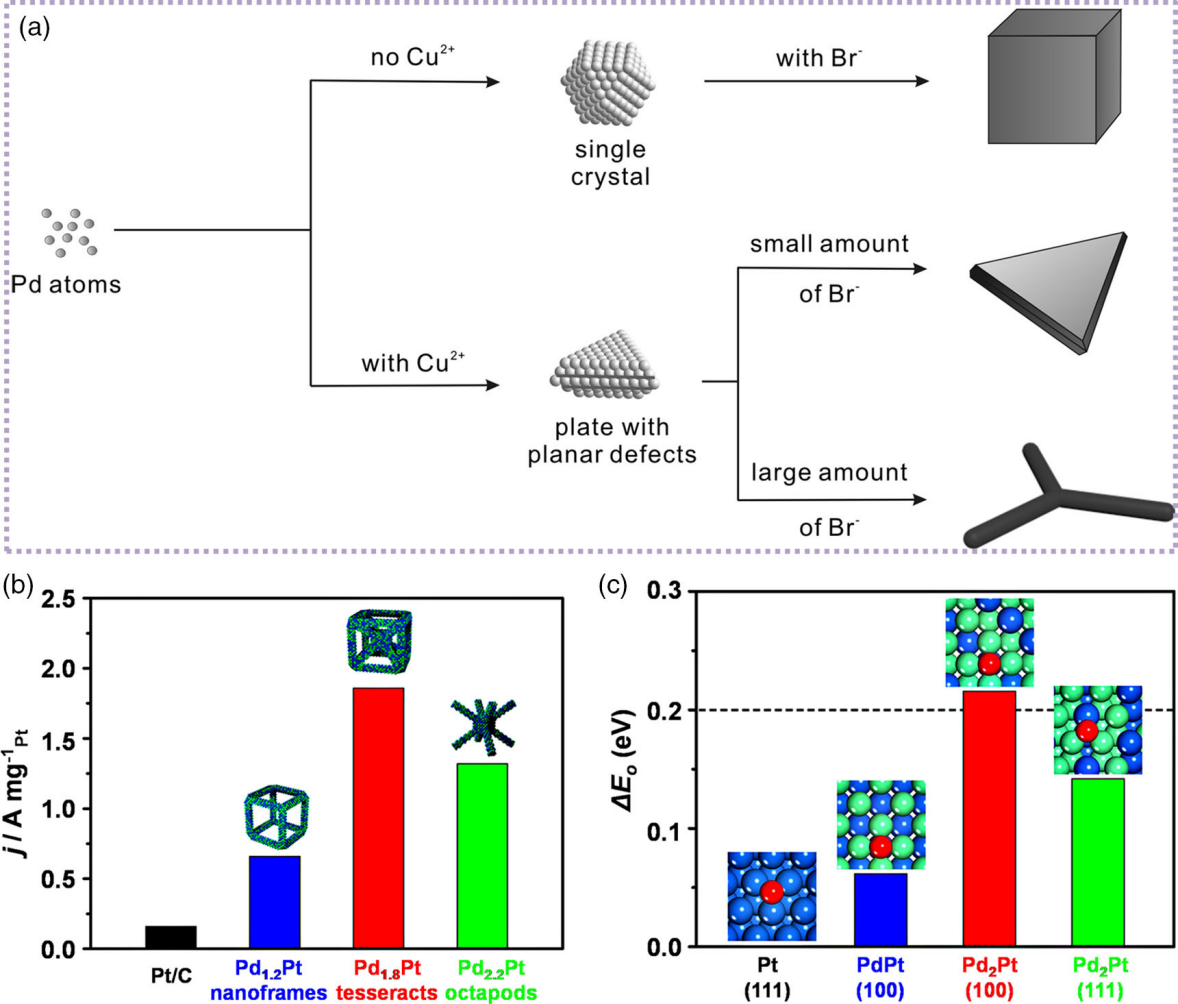
The 3D Pd‐based nanostructures for efficient electrocatalysis. a) Schematic illustrations showing the evolution of Pd nanocrystals with different morphologies as controlled by both Cu^2+^ and Br^−^ ions. Reproduced with permission.^[^ ^50^ ^]^ Copyright 2014, Wiley‐VCH. b) The ORR mass activities of different Pd—Pt catalysts at 0.9 V versus RHE and c) the calculated oxygen adsorption energy on different Pd—Pt surfaces relative to that on Pt (111) surface (the green, blue, and red spheres represent the Pd, Pt, and O atoms, respectively, and the short dashed horizontal lines represent the optimal Δ*E*_O_, 0.2 eV). b,c) Reproduced with permission.^[^ ^51^ ^]^ Copyright 2021, American Chemical Society.

Pd‐based alloyed nanocrystals with unique 3D structures attracted increasing attentions recently. In the case of nanodendrites, high‐quality PdCu bimetallic tripods were reported by controlling the nucleation way that the plate‐like crystal seeds were formed in the initial step and Pd atoms were forced to deposit onto the three corners of a seed.^[^ ^50^ ^]^ As shown in **Figure** **4**a, the Pd single‐crystal seeds can be obtained in the absence of Cu^2+^ to drive the formation of Pd nanocubes. However, the additional Cu^2+^ can induce the formation of plate‐like seeds due to the low stacking fault energy of Cu atoms. Finally, the Br^−^ can block the three (111) facets of each triangular seed, thus more KBr resulted in the formation of tripods. More recently, three different Pd—Pt hollow structures, including octapods, tesseracts, and nanoframes, were synthesized by selectively etching Pd—Pt nanocubes.^[^ ^51^ ^]^ Note that as‐obtained Pd—Pt tesseracts displayed the highest mass activity of 1.86 A mg^−1^, 11.6 times higher than that of commercial Pt/C, also much higher than those of Pd—Pt octapods and nanoframes (Figure 4b). The mechanistic studies indicated that the enhanced ORR activity of Pd—Pt tesseracts was mainly attributed to the facet and composition effect, resulting in an optimized oxygen adsorption energy (Figure 4c). Beyond doubt, combining the alloying strategy and morphology‐controlled synthesis, the adsorption of catalysts toward reaction intermediates was well tuned, providing the possibility to obtain enhanced electrocatalytic activity and durability.

For the other 3D Pd‐based nanostructures, the hierarchical Pd_4_Fe nanoflowers were synthesized by reducing Pd(acac)_2_ and Fe(CO)_5_ in the oleylamine.^[^ ^52^ ^]^ In this synthetic system, the adsorption of CO decomposed from Fe(CO)_5_ made the nanosheet petals expose (111) facets. When used for ORR electrocatalysis, the mass activity of Pd_4_Fe nanoflowers was 8 times higher than that of commercial Pd/C. The enhanced ORR activity can be attributed to the combination of unique hierarchical architectures and the alloying of Pd and Fe. In addition, to obtain Pd‐based multimetallic hollow mesoporous nanospheres, a simple and effective aqueous approach was presented to synthesize the trimetallic PdAgCu hollow mesoporous nanospheres.^[^ ^53^ ^]^ This kind of PdAgCu possessed adjustable interior hollow cavity and mesoporous shell. The unique structure was originated from the addition of dioctadecyldimethylammonium chloride (DODAC), a typical dual‐template structural directing surfactant. Several advantages, including multimetallic composition and the hollow and mesoporous structures, synergistically enhanced the EOR electrocatalytic performance.

Aerogels, as a kind of porous materials, show ultralow density, large surface area and profuse open interconnected pores, thus attracting increasing attentions in electrocatalytic applications.^[^ ^54^ ^]^ Although a large number of inorganic aerogels, such as graphene, metal oxides, and the corresponding complex systems, have been widely developed, unsupported noble metal‐based aerogels are still quite limited, especially Pd‐based multimetallic aerogels.^[^ ^55^ ^]^ For this purpose, the PdCu bimetallic aerogels were realized by boosting the gelation kinetics in aqueous solution, which is addressed by increasing the elevated temperatures.^[^ ^56^ ^]^ This synthetic method did not involve the additional capping agent, ensuring the clear surface of aerogels. The PdCu aerogels with well‐defined nanowire networks showed superior EOR performance due to the synergistic structural and compositional effects.

## 3 Advanced Regulation of Pd‐Based Nanomaterials for the Enhanced Electrocatalysis

**Scheme 2:**
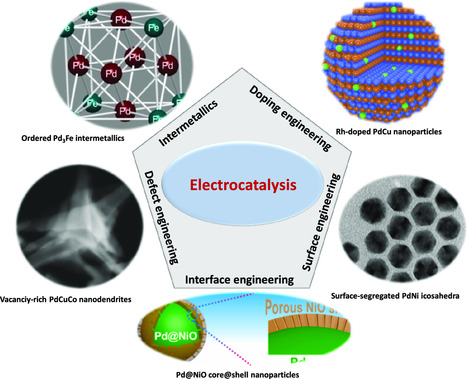
Illustration of key strategies for enhancing electrocatalytic performance of Pd‐based alloy electrocatalysts. Ordered Pd_3_Fe intermetallics: Reproduced with permission.^[^ ^13a^ ^]^ Copyright 2015, American Chemical Society. Vacancy‐rich PdCuCo nanodendrites: Reproduced with permission.^[^ ^13b^ ^]^ Copyright 2018, Wiley‐VCH. Surface‐segregated PdNi icosahedra: Reproduced with permission.^[^ ^13c^ ^]^ Copyright 2018, American Association for the Advancement of Science. Pd@NiO core@shell nanoparticles: Reproduced with permission.^[^ ^13d^ ^]^ Copyright 2019, Elsevier. Rh‐doped PdCu nanoparticles: Reproduced with permission.^[^ ^13e^ ^]^ Copyright 2020, Peking University Press.

Although the state‐of‐the‐art Pd‐based electrocatalysts have been achieved based on the structural and compositional regulation of Pd‐based nanoalloys, it is still difficult to break the ceiling of catalytic performance. However, the advanced designs of Pd‐based electrocatalysts have witnessed the unprecedented enhancement of electrocatalysis by the optimized electronic properties, modified active sites, and tunable adsorption ability. Benefiting from these advanced strategies, including intermetallics, doping, defects, surface, and interface engineering, Pd‐based multimetallic electrocatalysts have achieved significant advances (**Scheme** **2**).^[^ ^13^ ^]^ In this section, the achievements of high‐performance Pd‐based electrocatalysts are presented in detail based on the fundamental understanding of these regulation strategies. In addition, by presenting key electrocatalytic reactions, we demonstrated the significance of different strategies in the enhanced electrocatalysis.

### 3.1 Intermetallics

Generally, the conventional alloyed Pd‐based electrocatalysts possess a random distribution of atomic positions, thus inducing a poor chemical and structural stability. In contrast, intermetallic structures, where the determined locations are accurately occupied by constituent atoms in an ordered fashion, can provide strong structural robustness due to higher mixing enthalpy and stronger atomic interaction between Pd and other metal atoms (M, usually Cu, Fe, Pb, etc.).^[^ ^57^ ^]^ Therefore, these Pd‐based intermetallic catalysts can display the enhanced activity and durability.

**Figure 5:**
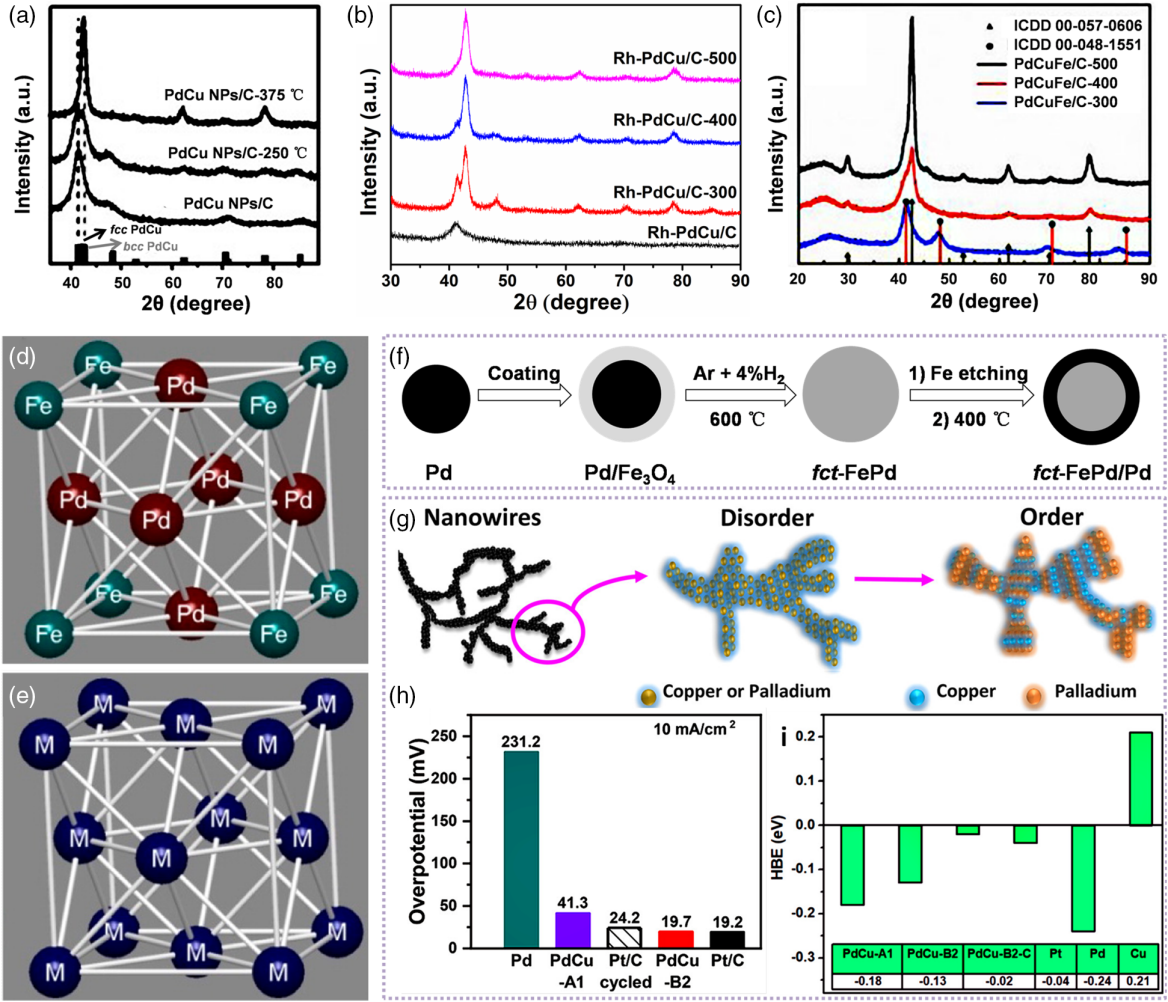
The typical strategy for enhancing electrocatalytic performance of Pd‐based nanoalloys by intermetallics obtained from posttreatment process. PXRD patterns of different carbon‐supported a) PdCu nanoparticles (PdCu NPs/C), b) Rh‐doped PdCu nanoparticles (Rh‐PdCu/C), and c) PdCuFe nanoparticles (PdCuFe/C) at different thermal temperatures. a) Reproduced with permission.^[^ ^58^ ^]^ Copyright 2016, Wiley‐VCH. b) Reproduced with permission.^[13e]^ Copyright 2020, Peking University Press. c) Reproduced with permission.^[^ ^59^ ^]^ Copyright 2019, Wiley‐VCH. Crystal structures of d) ordered Pd_3_Fe intermetallics and e) disordered Pd_3_Fe alloys. d,e) Reproduced with permission.^[13a]^ Copyright 2015, American Chemical Society. f) Schematic illustration of the synthesis of core/shell fct‐PdFe/Pd nanoparticles. Reproduced with permission.^[^ ^60^ ^]^ Copyright 2015, American Chemical Society. g) Scheme for the synthesis of the ordered PdCu intermetallic nanowire networks, h) HER overpotential at a current density of 10 mA cm^−2^ in 0.5 m H_2_SO_4_ and i) HBEs calculated from DFT of ordered and disordered PdCu nanowire networks, commercial Pd/C and Pt/C catalysts (PdCu‐A1 and PdCu‐B2 represent the random A1‐phased and intermetallic B2‐phase PdCu alloys, respectively). g–i) Reproduced with permission.^[^ ^64^ ^]^ Copyright 2020, American Chemical Society.

An important route for synthesizing intermetallics is high‐temperature‐induced phase transformation of the disordered or core–shell counterparts. A series of ordered PdCu‐based intermetallic nanoparticles (PdCu, PdCuCo, and PdCuNi) were successfully prepared via annealing the disordered counterparts at a temperature of 375 °C (**Figure** **5**a).^[^ ^58^ ^]^ However, when introducing Rh or Fe metals, the phase transition temperature was increased to 500 °C, indicating that the Rh and Fe elements can inhibit the phase transformation of PdCu nanoparticles (Figure 5b,c).^13^, ^59^ Due to the strong interatomic interaction, these intermetallic nanoparticles showed the enhanced performance in many electrocatalytic reactions, such as ORR and EOR. In terms of other Pd‐based intermetallics, Cui et al. explored the annealing conditions controlled the ordered behavior of Pd_3_Fe nanoparticles.^[13a]^ The KCl matrix generated from potassium triethylborohydride (KEt_3_BH) can prevent the agglomeration of the samples during thermal process. The disordered Pd_3_Fe nanoparticles were obtained under an annealing temperature of 400 °C, while the ordered Pd_3_Fe intermetallics were prepared at 600 °C. As‐obtained products annealed at 600 °C showed an ordered crystal structure, where both Pd and Fe atoms occupied their own positions, whereas the atomic positions were distributed randomly as for the disordered counterparts (Figure 5d,e). Compared with the disordered counterpart, this ordered Pd_3_Fe intermetallics showed the higher ORR activity and durability in alkaline media.

To further facilitate the interatomic diffusion within a nanoparticle, core/shell structural precursors were selected to transfer to be intermetallics. Intermetallic face‐centered‐tetragonal (fct) structural PdFe nanoparticles were synthesized by annealing core/shell Pd/Fe_3_O_4_ nanoparticles at 600 °C under a reducing atmosphere (4% H_2_/Ar).^[^ ^60^ ^]^ The core/shell fct‐PdFe/Pd nanoparticles were obtained by etching surface Fe atoms (Figure 5f). By rationally tuning the thickness of Pd shell, the nanoparticles with 0.65 nm shells showed the highest Pt‐like ORR activity under acidic conditions. The enhanced performance was mainly originated from the desired Pd lattice compression for Pd shell. Another method for facilitating metal atom interdiffusion is the incorporation of foreign metal atoms with weaker alloying ability with Pd. As the strong alloy interaction between Pd and second metal atoms can extrude the foreign atoms onto the surface of nanocrystals, the vacancy can be left to drive the interdiffusion of atoms, forming an intermetallic structure. Inspired by this strategy, core/shell‐structured PdCo nanoparticles were transferred into ordered intermetallic phases by incorporating additional Au atoms.^[^ ^61^ ^]^ The introduction of Au atoms not only addressed the synthetic challenge of intermetallic PdCo nanoalloys, but also provided a strong protective effect for the structural change. Benefiting from the atomic structural ordering and the protective effect of Au atoms, the ordered AuPdCu nanopartiles showed the enhanced ORR performance, especially the significantly improved durability. In addition, thermal annealing‐induced Pd‐based intermetallics have also been realized in many ordered intermetallic Pd‐based nanocrystals, such as PdPb, PdIn, PdSn, and PdZn.^[^ ^62^ ^]^

Although various Pd‐based intermetallics with precise composition have been successfully prepared by thermal annealing, it is difficult to synthesize the intermetallic nanocrystals with controlled morphology. Therefore, it is imperative to synthesize the Pd‐based intermetallics in a mild and simple method. To obtain PdCu intermetallic nanocubes, PdCl_2_, Cu(acac)_2_, and trioctylphosphine (TOP) were served as metal precursors and stabilizer to promote the formation of PdCu nanocubes, respectively.^[^ ^24^ ^]^ The halide ions played an important role in controlling the ordered metallic structure, and the amounts of TOP influenced the morphology of nanocrystals. By changing the precursor salts to chloride counterparts, PdCu intermetallics can also be synthesized through a simple wet‐chemical approach, confirming the significance of halide ions in the formation of intermetallic structures.^[^ ^63^ ^]^ The amount of triphenylphosphine (TPP), serving as capping reagent, can control the morphology of PdCu intermetallics. A small amount of TPP induced the formation of PdCu nanocubes, while increasing the amount of TPP can form the spherical PdCu nanoparticles. In addition, the PdCu nanowire networks with intermetallic structures were transformed from alloyed disordered PdCu nanowires through an electrochemical treatment strategy (Figure 5g).^[^ ^64^ ^]^ The intermetallic PdCu nanowire networks exhibited lower HER overpotential and higher exchange current density than that of commercial Pt/C (Figure 5h). DFT calculations revealed that the decreased hydrogen binding energy (HBE) was obtained on the compressed intermetallic PdCu nanowires, in which an optimal position of HBE close to Pt catalysts was found (Figure 5i). Inspired by the ordering process occurring with the fact that the nanocrystals need to overcome a size‐dependent activation barrier, the ideal model of intermetallics is available for studying the electrocatalytic structure–performance relationship.

**Figure 6:**
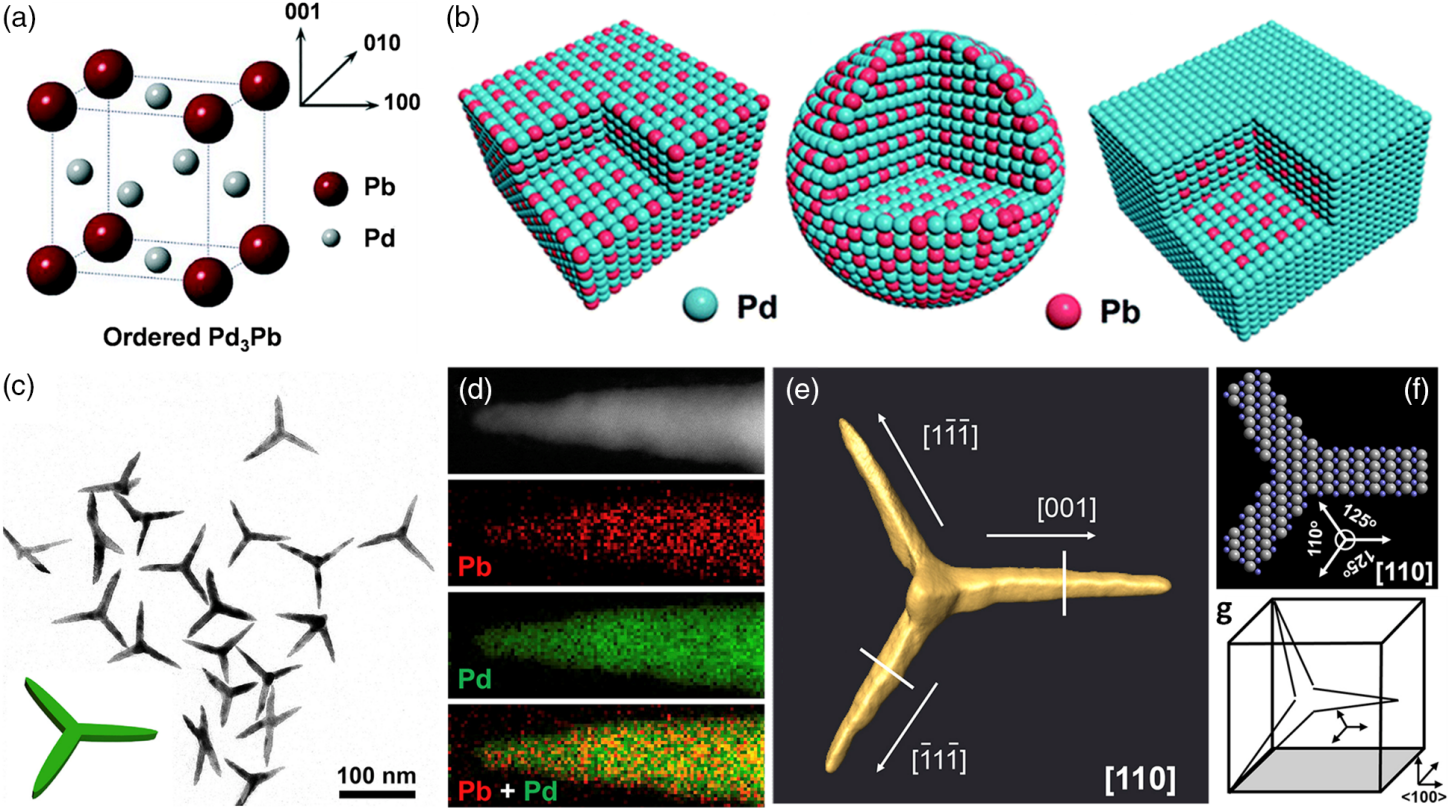
The typical strategy for enhancing electrocatalytic performance of Pd_3_Pb intermetallics. a) Schematic diagram of the crystal structure of the ordered Pd_3_Pb intermetallic phase with a Cu_3_Au structure type. Reproduced with permission.^[^ ^65^ ^]^ Copyright 2019, The Royal Society of Chemistry. b) Schematic illustration of morphologies and surfaces of different intermetallic Pd_3_Pb nanocrystals, including Pd_3_Pb nanocubes, Pd_3_Pb nanoparticles, and Pd‐skin Pd_3_Pb nanocubes from left to right. Reproduced with permission.^[67b]^ Copyright 2019, The Royal Society of Chemistry. c) TEM image, d) corresponding STEM‐EDS elemental mapping, e) reconstructed 3D tomograms along the [110] zone axis, f) atomic model along the [110] zone axis, and g) 3D illustration along the <100> zone axis of ordered intermetallic Pd_3_Pb nanotripods. c–g) Reproduced with permission.^[^ ^69^ ^]^ Copyright 2018, Elsevier.

Compared with the other Pd‐based intermetallics, Pd_3_Pb intermetallics with structured morphology are much easier to synthesize. This is because that the ordered Cu_3_Au structure‐type Pd_3_Pb is more thermodynamically stable than the disordered counterparts. Generally, a unit cell of Pd_3_Pb intermetallics contains Pd atoms occupied the center and Pb atoms located at the corner positions (**Figure** **6**a).^[^ ^65^ ^]^ Such periodic atomic arrangement makes the ordered Pd_3_Pb intermetallics be highly active due to the geometric and electronic effect, as well as the bifunctional mechanism.^[^ ^66^ ^]^ To further promote the electrocatalytic activity, Pd_3_Pb intermetallics with various morphologies, such as nanocubes, nanowires, and nanosheets, were designed by simple and mild wet‐chemical methods.^[^ ^67^ ^]^ Various intermetallic Pd_3_Pb nanocrystals, including Pd_3_Pb nanocubes, Pd_3_Pb nanoparticles, and Pd‐skin Pd_3_Pb nanocubes, were selectively designed in a mixed oleylamine/1‐octadecene system (Figure 6b).^[67b]^ Mandelic acid and 1‐octadecene induced the formation of nanocube morphology, and excess Pd salts generated Pd‐skin structure. By replacing the mandelic acid with l‐ascorbic acid, however, the morphology of nanocubes converted into nanoparticles. Compared with the low‐dimensional nanocrystals, the 3D nanobranched structures play a crucial role in improving electrocatalytic activity and durability.^[^ ^68^ ^]^ For this purpose, Bu et al. proposed a strong coupled *s—p—d* exchange effect‐based onordered intermetallic Pd_3_Pb tripods with predominantly (110) facets for highly active and stable ORR electrocatalysis.^[^ ^69^ ^]^ A chemical yield of ≈90% was achieved through using acetylacetonates as precursor salts, phloroglucinol as reducing agent, and mixed oleylamine/oleic acid as surfactants and solvents (Figure 6c,d). For a single Pd_3_Pb tripod, two branches possessed <111>‐type growth directions and the third one was (001) orientated (Figure 6e–g), opening up a new design concept for high‐dimensional ordered Pd‐based intermetallics. In this catalysts system, the strong charge exchange between Pd‐4*d* and Pb‐(*sp*) orbital would be happened on the Pd_3_Pb (110) facets. As a results, the Pd_3_Pb tripod exhibited unprecedented ORR activity and durability.

### 3.2 Doping

Doping refers to an effective strategy of intentionally incorporating a small amount of foreign atoms into the lattice of the host Pd or Pd‐based nanocrystals. This strategy can efficiently change the electronic structure of the host electrocatalysts, thus beneficial for the enhanced electrocatalysis. Various elements with positive effects toward different electrocatalytic reactions have been successfully doped into Pd‐based electrocatalysts, such as Ir, Fe, W, Rh, and Au.^13^, ^62^, ^70^ For precisely controlling the surface chemical and electronic environments, these different dopants and the doping concentrations can be well recognized in the host catalysts by several methods, including wet‐chemical method or two‐step seed‐mediated growth approach.

**Figure 7:**
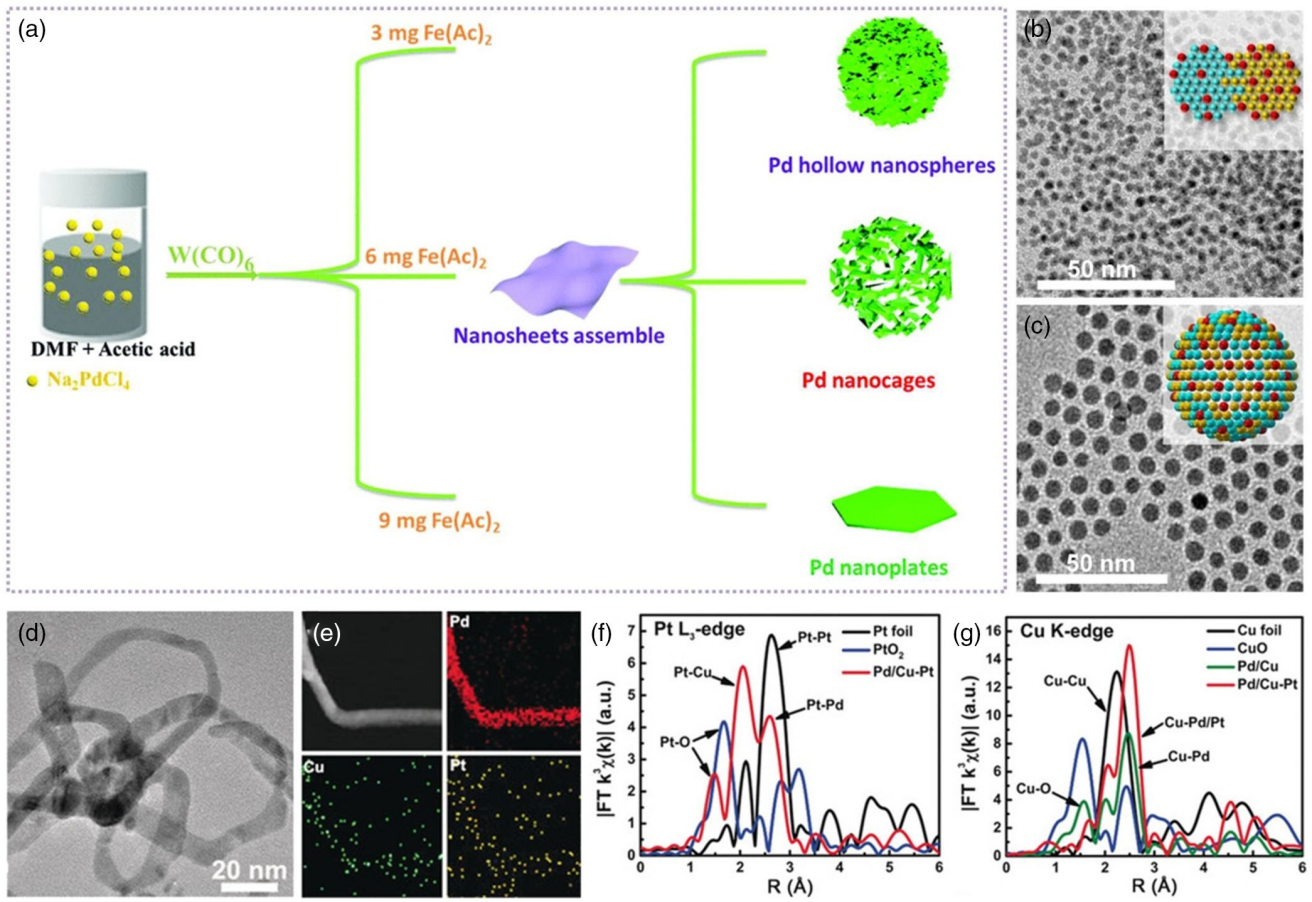
The typical strategy for enhancing electrocatalytic performance of Pd‐based nanoalloys by doping engineering. a) Schematic illustration of the synthesis and the corresponding TEM images of PdFe hollow nanospheres, PdFe nanocages, and PdFe nanoplates. Reproduced with permission.^[70d]^ Copyright 2020, The Royal Society of Chemistry. TEM images of Pd—Ni—P nanoparticles with shortened distance between Pd and Ni active sites by different phosphorization conditions of b) 260 °C, 5 min (Pd_38_Ni_49_P_13_) and c) 260 °C, 1 h following 290 °C, 1 h (Pd_40_Ni_43_P_17_). b,c) Reproduced under the terms of the CC‐BY 4.0 license.^[^ ^74^ ^]^ Copyright 2017, The Authors, published by Springer Nature. d) High‐magnification TEM image, e) corresponding STEM‐EDS elemental mapping, the *k*_3_‐weighted *χ*(*k*)‐function of the EXAFS spectra for the f) Pt L_3_‐edge and g) Cu K‐edge of Cu—Pt dual sites alloyed with Pd nanorings (Pd/Cu—Pt NRs). d–g) Reproduced with permission.^[^ ^79^ ^]^ Copyright 2017, Wiley‐VCH.

One of the most straightforward strategies for synthesizing the foreigner atoms‐substituting alloy catalysts is the facile wet‐chemical approach, which is to directly reduce the stoichiometric metal precursors in the well‐proportioned liquid phase. By using a one‐pot wet‐chemical method, Rh‐doped PdAg nanoparticles with highly active and stable ORR performance and great methanol tolerance ability were successfully synthesized.^[70b]^ Based on the DFT results, the chosen doping element (Rh), providing abundant 4*d* orbital energy levels, can serve as the active bridge to connect the surface Ag and Pd electronic states. Therefore, high‐efficiency adsorption/desorption was realized by downshifting *d*‐band centers. In the tuned *d*‐electron downshifting potential process, the ORR intrinsic barriers were alleviated. Therefore, the ORR performance of PdAg nanoparticles was significantly enhanced by the Rh‐doping strategy. Meanwhile, the Rh‐doped PdAg nanoparticles also showed great methanol tolerance capability. The current results emphasized the important role of Rh atoms in Pd‐based methanol tolerance ORR electrocatalysts. The similar experimental phenomenon was further observed in the ordered intermetallic Rh‐doped PdCu nanoparticles.^[13e]^ 2D ultrathin Pd‐based nanomaterials, representing a class of novel structures for high‐performance electrocatalysis, have received considerable interests in heterogeneous catalysis. This is due to the unique electronic and surface properties, which are different from their counterparts in other dimensions. Doping the foreigner atoms into the ultrathin nanocrystals has the potential to further magnify the structural advantages for promising electrocatalytic performances. Porous Fe‐doped Pd nanosheets assemblies were prepared via the coreduction of Na_2_PdCl_4_ and Fe(CH_3_COOH)_2_ in the mixed N,N‐dimethylformamide (DMF) and acetic acid solvents under the assistance of W(CO)_6_ and polyvinylpyrrolidone (PVP).^[70d]^ By varying the feeding amount of Fe(CH_3_COOH)_2_, several different Fe‐doped Pd nanosheets assemblies with a morphological modulation from hollow nanospheres, nanocages to nanoplates were obtained (**Figure** **7**a). The incorporation of Fe atoms modified the initial electronic structure of Pd to downshift the *d*‐band center of Pd, beneficial for the adsorption of intermediates. Therefore, the optimized Fe‐doped Pd nanosheet assemblies showed much improved MOR and EOR electrocatalysis compared with the other controlled catalysts.

Two‐step seed‐mediated growth approach tends to obtain surface‐doped catalysts through galvanic replacement or substitution methods on a designated alloyed platform. Using the Pd nanocubes as growth seeds, well‐defined W‐doped Pd nanocubes with regular surface structures were prepared by the surface‐doping strategy.^[70a]^ The atomic ratio of surface W atoms was controlled by tuning the feeding amount of W(CO)_6_, and the optimized 1.2% W‐doped catalysts exhibited the best ORR performance. The varied electronic structure of Pd atoms caused by W‐doping downshifted the *d*‐band center and optimized the adsorption ability of the intermediates, thus achieving the enhanced ORR activity. To obtain ordered Au‐doped PdZn intermetallics, a galvanic replacement approach was performed to substitute Pd or Zn atoms in presynthesized ordered intermetallic PdZn nanoparticles with Au atoms.^[62d]^ After the modification of Au atoms, the intermetallic structures were well maintained. The strong interaction between PdZn and doped Au species endowed the catalysts more excellent ORR performance compared with the undoped counterparts. Therefore, it is anticipated to choose more positive elements to be incorporated into Pd‐based nanocrystals to enhance electrocatalytic activity and durability.

In addition to metal elements, several nonmetallic species, such as H, B, S, P, and Se, have also been introduced into Pd‐based alloys to improve the electrocatalytic performance.^[^ ^71^ ^]^ The doping of nonmetallic atoms visualizes several advantages for facilitating the adsorption of oxygen‐related species during electrocatalytic reactions.^[^ ^72^ ^]^ First, the nonmetallic atoms with abundant valence electrons can provide more additional adsorption sites to accelerate the reaction kinetics. In addition, the formation of Pd oxides can be weakened by enhancing the oxidation potential, and the electronic structure can be further modified to balance the adsorption/desorption of intermediates. To understand how B‐doping enhanced the ORR activity of pure Pd catalysts, the adsorption ability of ORR intermediate species between Pd and B‐doped Pd nanoalloys was compared by combining the DFT calculations and experimental results.^[^ ^73^ ^]^ Based on the theoretical models, the negatively shift of the surface core level of Pd atoms implied that the additional electrons derived from incorporated B atoms filled in the states density related to the antibonding of oxygen. Therefore, the absorption of oxygen‐related intermediates with nearly optimal binding energy was weakened, which facilitated the ORR electrocatalysis. This viewpoint can be further confirmed by the fact that as‐synthesized B‐doped Pd nanoparticles showed superior ORR performance to pure Pd catalysts. The EOR activities of PdNi catalysts were highly relevant to the distance between Pd and Ni active sites, which can be abbreviated by doping P into PdNi nanoparticles.^[^ ^74^ ^]^ By tailoring the phosphorization conditions, including temperatures and times, the phase‐segregation Pd/Ni—P heterodimers were further converted into Pd—Ni—P alloyed nanoparticles with closer Pd—Ni distance (Figure 7b,c). Due to the shortened distance between Pd and Ni active sites, the formation of free ·OH radicals was significantly accelerated. Therefore, the following combination of ·OH and CH_3_CO· radicals, representing the rate‐determining step of EOR, was boosted. By increasing the active sites of noble and oxophilic metals in multicomponent catalysts, this study revealed a novel nonmetal‐doping method to enhance the electrocatalytic activity and durability.

Single atom alloy, as an emerging atomic‐sites catalyst, has been developed to depict the unique doping systems, mainly involving single clusters and single atom catalysts.^[^ ^75^ ^]^ The design of single atom alloy involves a low‐content component in the host alloy catalysts isolated from each other, thus producing free atom‐like electronic structures.^[^ ^76^ ^]^ In the single atom alloy, the altered electronic structures can influence the activation of reaction molecules and the adsorption of intermediates, thereby boosting the overall catalytic activities. A typical example is that the ultrathin Pd nanosheets can be broken off to transform into ultrathin Pd nanoribbons with abundant bends and kinks after adding a small amount of RuCl_3_·*x*H_2_O into the as‐formed Pd nanosheets.^[^ ^77^ ^]^ The atomically dispersed isolated Ru atoms were anchored onto the steps and edges of the non‐atomically flat Pd nanoribbons. Single atom Pd active site can serve as a possible candidate to reduce O_2_ to H_2_O_2_ with high selectivity. This can be further confirmed by the recent results that the dispersed Pd single atoms on the surface of Au can well reduce oxygen to H_2_O_2_.^[^ ^78^ ^]^ A more interesting study is that using the atomically dispersed Cu on ultrathin Pd nanorings as growth seeds to demonstrate the formation of Cu—Pt dual sites alloyed with Pd nanorings by sequential reduction strategy (Figure 7d,e).^[^ ^79^ ^]^ Extended X‐ray absorption fine structure (EXAFS) characterizations revealed that a dominating peak located at 2.5 Å for Cu K‐edge spectra, corresponding to the Cu—Pt or Cu—Pd coordination (Figure 7f). This result illustrated that the Cu sites were atomic dispersion. After introducing a small amount of Pt atoms, Cu—Pt dual sites single atom alloy was formed. The Pt atoms were homogeneously dispersed on Pd/Cu nanorings (Figure 7g). The enhanced HER activity was originated from that the Cu atom nearing the Pt sites can balance the interactions between the H atoms and Pt atoms, providing a new idea for designing high‐performance electrocatalysts at an atomic level.

### 3.3 Defects

**Figure 8:**
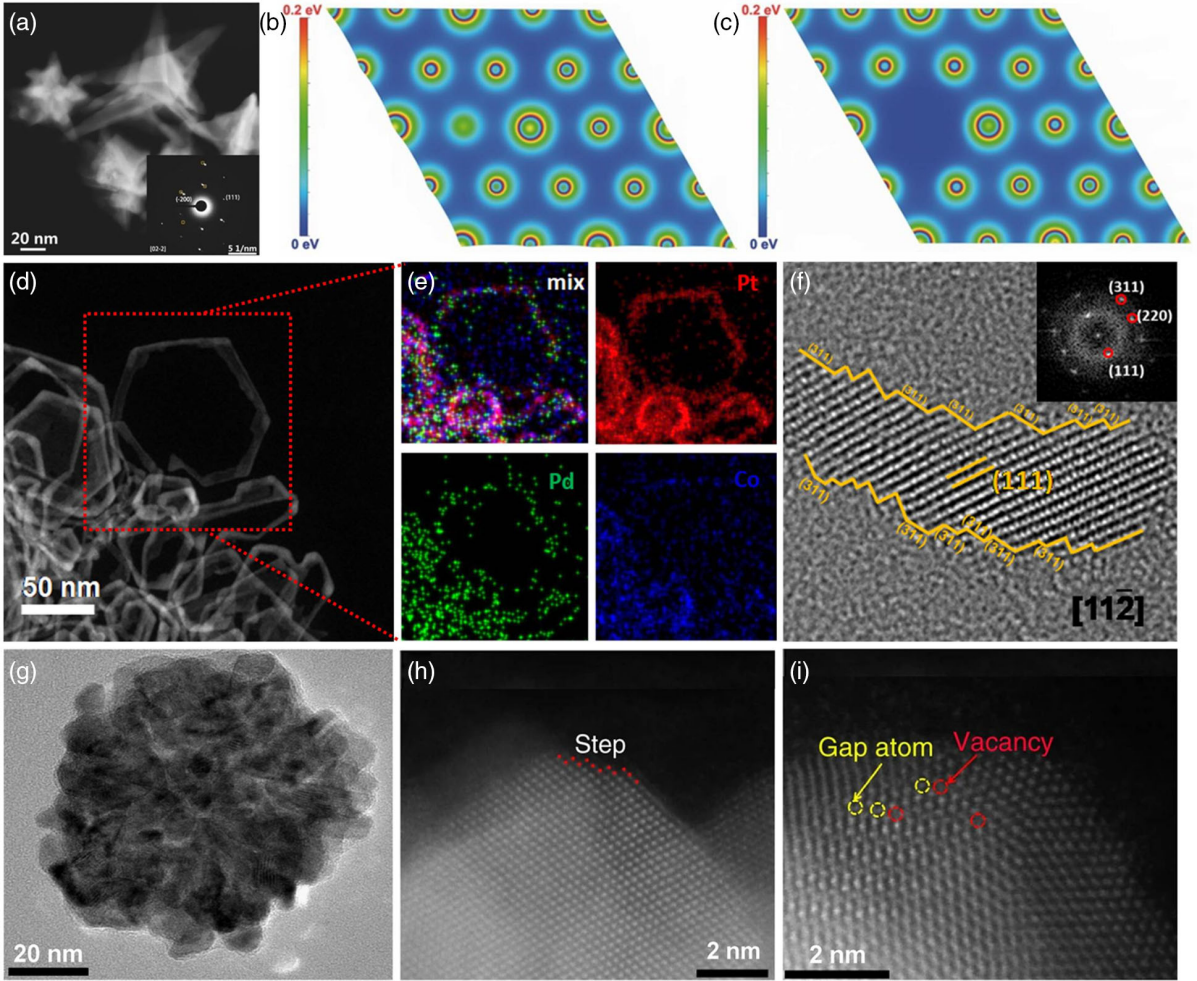
The typical strategy for enhancing electrocatalytic performance of Pd‐based nanoalloys by defect engineering. a) HAADF‐STEM image of the vacancy‐rich PdCuCo anisotropic structure, the surface charge density of b) the Co‐embedded PdCu anisotropic structure, and c) the vacancy‐rich PdCuCo anisotropic structure. a–c) Reproduced with permission.^[13b]^ Copyright 2018, Wiley‐VCH. d) HAADF‐STEM image, e) corresponding STEM‐EDS elemental mapping, and f) high‐resolution TEM (HRTEM) image along the zone axes of [112] axis (inset is the corresponding fast Fourier transform (FFT) pattern) of PtPdCo nanorings. d–f) Reproduced with permission.^[^ ^82^ ^]^ Copyright 2018, Wiley‐VCH. g) High‐magnification TEM image, aberration‐corrected TEM images for h) surface steps and i) gap atoms in yellow dashed circle and vacancy in red dashed circle of Pd_59_Cu_30_Co_11_ nanodendrites. g–i) Reproduced under the terms of the CC‐BY 4.0 license.^[^ ^84^ ^]^ Copyright 2018, The Authors, published by Springer Nature.

The defect engineering is an effective strategy to modulate the adsorption/desorption behaviors of catalysts toward reaction intermediates.^[^ ^80^ ^]^ By designing defect structures, such as point, line, and plane defects, the varying localized crystal structure can be realized to induce the enhancement of electrocatalytic performance. Focusing on the abundant vacancy defects, the vacancy‐rich multicomponent PdCuCo anisotropic structure was reported as a desired platform to polarize the surface structures of PdCuCo anisotropic structure (**Figure** **8**a–c).^[13b]^ Specifically, the incorporation of Co atoms induced a significant difference of crystal structure between the PdCu and PdCuCo anisotropic structures. Benefitting from the atomic rearrangement caused by the incorporation of Co atoms, the vacancies on the surface provided more favorable spatial position for releasing the strain effect. Thus, the ORR activity was significantly improved.

More extensive research interests are concentrated on the step edges, which can strongly affect the coordination environments of catalysts surface. By the controllable etching approaches, several kinds of low‐dimensional Pd nanostructures with tailored fractal edge sites can be synthesized, including nanobelts, porous nanosheets, and nanoframes.^[^ ^81^ ^]^ The unique structural and chemical features of the edge sites can serve as catalytic active centers to drive high‐performance FAOR. Another important example is PtPd‐based nanorings (including PtPdCo, PtPdFe, and PtPdNi), in which the abundant stepped atoms played crucial roles in the optimized adsorptive behaviors of oxygen catalytic intermediates (Figure 8d–f).^[^ ^82^ ^]^ To generate the surface dominated by the high‐quality step atoms, the oxidation etching by O_2_ under the presence of Br^−^ was essential. More recently, PdPtCu trimetallic nanorings with in situ enriched low‐coordinated edges sites were reported for the vigorously enhanced C—C bond cleavage ability of EOR using the similar strategy.^[^ ^83^ ^]^ Based on these results, the “metal‐edge‐driven” concept was formally presented to promote the electrocatalytic activity. The surface electronic structure of PdCuCo trimetallic dendritic nanocrystals was also optimized by combining the defects with surface strain.^[^ ^84^ ^]^ The low‐coordinated atoms situated on the abundant defects, forming during in situ synthesis process, were well observed on the surface of trimetallic nanocrystals (Figure 8g–i). As‐synthesized dendritic structures provided the high activity toward ORR, MOR, and FAOR multifunctional electrocatalysis. Needless to say, adjusting the enrichment density of step edges can be considered as an effective strategy to regulate the *d*‐bond center for facilitating electrocatalytic performance.

**Figure 9:**
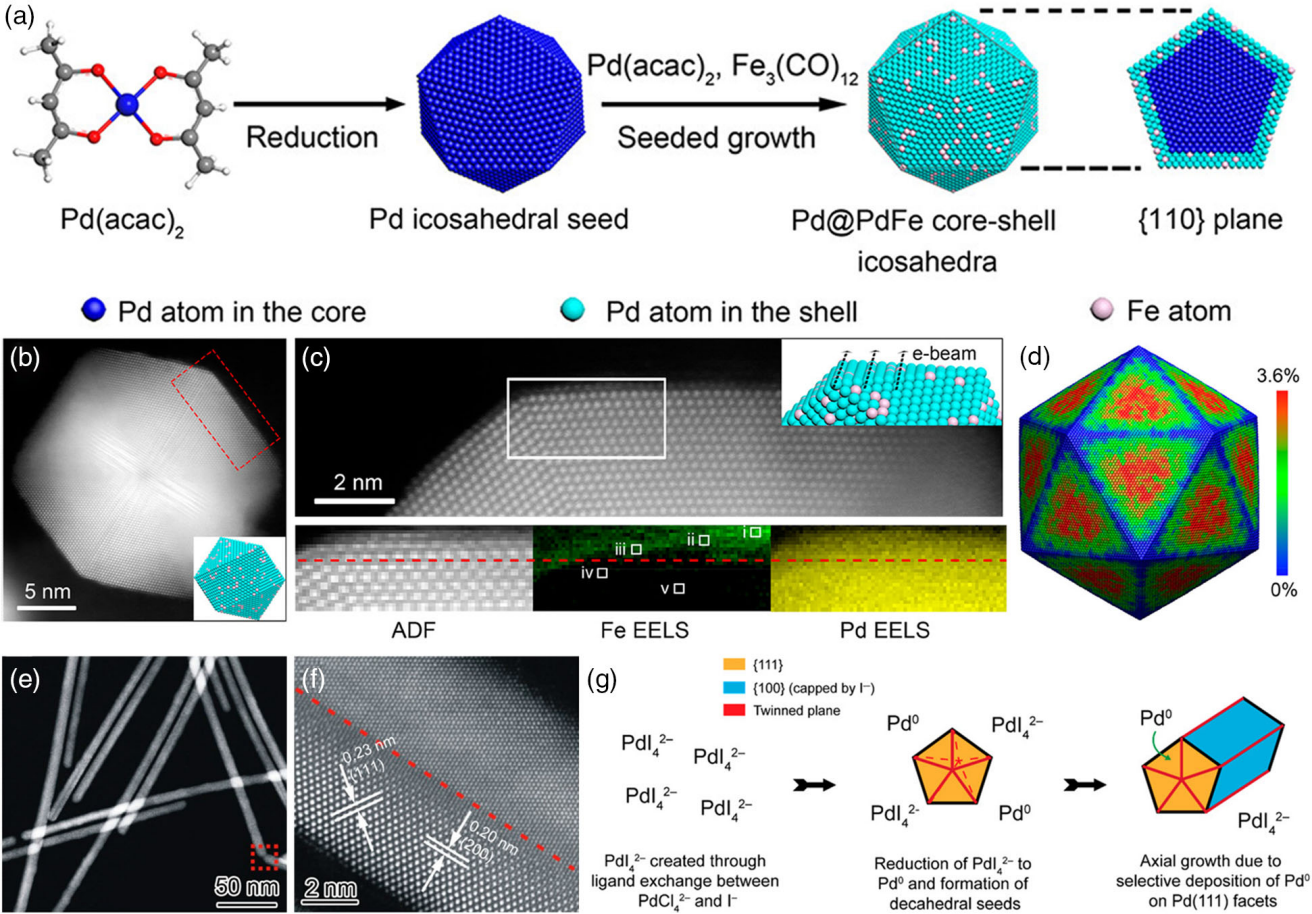
The typical strategy for enhancing electrocatalytic performance of Pd‐based nanoalloys by multitwinned structures. a) Schematic illustration of the synthesis of Pd/PdFe core/shell icosahedra, b) atomic‐resolution HAADF‐STEM image, c) HAADF‐STEM image at the edge region taken from (b) and the atomic‐resolution electron energy‐loss spectroscopy (EELS) mapping images of the Fe (green) and Pd (yellow), as well as a simultaneously acquired ADF image and d) surface strain field obtained from molecular dynamics (MD) simulations (color indicates strain labeled in the color map) of the Pd/PdFe core/shell icosahedra. a–d) Reproduced with permission.^[^ ^89^ ^]^ Copyright 2020, American Chemical Society. e) HAADF‐STEM image, f) HRTEM images of the region marked by a dashed box in (e), and g) schematic illustration of the nucleation and growth pathway for the formation of the penta‐twinned Pd nanowires. e–g) Reproduced with permission.^[^ ^91^ ^]^ Copyright 2017, American Chemical Society.

The multitwinned structure, representing an archetypal non‐0D defect, also possesses the unique influence on regulating the catalytic performance of Pd‐based materials.^[^ ^85^ ^]^ The multitwinned nanocrystals with the typical twin defects are mainly reflected in decahedra, icosahedra, and various 1D nanostructures with pentagonal cross sections. In these nanocrystals, microstrain is often expressed due to the deviation of the initial atoms from the ideal sites.^[12c]^ Among these different multitwinned nanocrystals, the icosahedral nanocrystals particularly exhibit high symmetry and optimized surface energy potential, thus changing the geometric and electronic structures of single‐crystal counterparts.^[^ ^86^ ^]^ However, due to the surface instability of the icosahedron and the oxidative etching of the twin defects under the presence of dissolved oxygen, Pd‐based icosahedral nanocrystals are difficult to precisely synthesize with a highly uniformity and purity. For this purpose, several kinds of capping agents, such as citrate ions, polyallylamine, and PVP, have been found to control the synthesis kinetics.^[^ ^87^ ^]^ These capping agents can usually protect the twin defects on the surface of the nanocrystals or changing the reduction potential of Pd precursors, inducing the formation of high‐quality icosahedra. Utilizing the PVP as a stabilizer and the citric acid as a reductant, the Pd icosahedra were synthesized in a high yield.^[^ ^88^ ^]^ In this synthesis system, the citric acid or citrate ions blocked the oxidation etching and ensured the formation of high‐quality multiply twinned Pd particles. Furthermore, the Pd icosahedra can be served as the core to heteroepitaxially form Pd/PdFe core/shell icosahedra.^[^ ^89^ ^]^ The three‐atomic‐layer tensile‐strained PdFe shell was formed by reducing Pd(acac)_2_ and decomposing Fe_3_(CO)_12_ (**Figure** **9**a). Due to the lattice mismatch between Pd cores and PdFe shells, an enlarged (111) plane was observed, inducing an average tensile strain of 1.89% (Figure 9b–d). Benefitting from the synergy of tensile strain and alloy effects, the binding energy for reaction intermediates was optimized. Therefore, a maximum ORR activity was achieved on the Pd/PdFe core/shell icosahedra. The multitwinned features can also be found in various 1D nanostructures, including nanowires and nanorods.^[^ ^90^ ^]^ By the combination of diethylene glycol and ascorbic acid to reduce Pd precursors, the Pd penta‐twinned nanowires were designed and showed an average diameter as thin as 7.8 nm and aspect ratios up to 100 (Figure 9e,f).^[^ ^91^ ^]^ The key for obtaining the twinned Pd nanowires was the regulation of reduction rate by controlling the feeding amount of NaI and HCl, which can complex with Pd atoms to form the PdI_4_^2—^ and suppress the dissociation of ascorbic acid. Another critical point was the presence of I^—^ that can selectively cap the Pd (100) surface, promoting the growth of the final products along the 1D direction to form multitwinned nanowires (Figure 9g). These multitwinned Pd‐based nanocrystals showed greatly enhanced catalytic activity than pristine Pd‐based catalysts among various electrochemical reactions.

### 3.4 Surface Engineering

Surface, an important position the catalytic reactions happen on, is highly relevant to the electrocatalytic properties.^[^ ^92^ ^]^ This is because that the surface structure can influence the interaction between the catalysts and reaction intermediates, thus optimizing the adsorption energy and activation energy of catalysts. Therefore, constructing the specific surface or subsurface is of significance for promoting the electrocatalytic performance, which can generally be realized by chemical etching, electrochemical dealloying, atmosphere annealing, and so on.^[^ ^93^ ^]^

**Figure 10:**
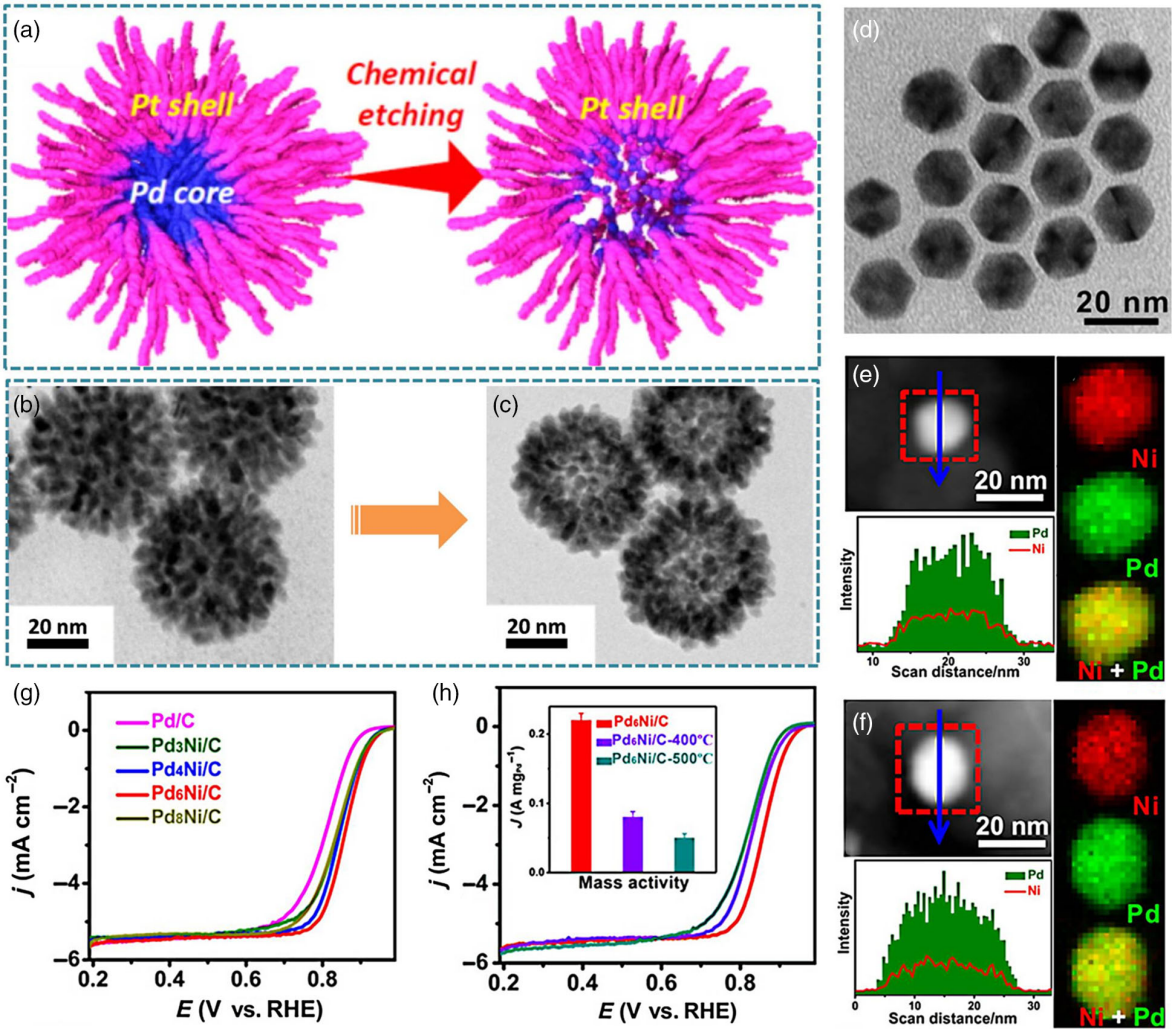
The typical strategy for enhancing electrocatalytic performance of Pd‐based nanoalloys by surface engineering. a) Schematic illustration for the formation of Pd/Pt dendritic nanocages with hollow interior and porous dendritic wall, TEM images of Pd/Pt nanodendrites b) before and c) after chemical etching. a–c) Reproduced with permission.^[^ ^94^ ^]^ Copyright 2013, American Chemical Society. d) TEM image of Pd_6_Ni icosahedra, HAADF‐STEM image, corresponding elemental mappings and line‐scan analysis of Pd_6_Ni icosahedra annealing at e) 400 °C and f) 500 °C. ORR polarization curves (a scan rate of 10 mV s^−1^ and a rotating rate of 1600 rpm in O_2_‐saturated 0.1 m KOH) of g) Pd—Ni icosahedra with different compositions and commercial Pd/C and Pt/C and h) Pd_6_Ni icosahedra with different annealing temperatures. d–h) Reproduced with permission.^[13c]^ Copyright 2018, American Association for the Advancement of Science.

The chemical etching method is a class of typical strategy for precisely regulating the surface environment of catalysts by controlling the etching strength. The active metal atoms can be removed from alloyed nanocrystals, resulting in the formation of novel concave/hollow structures. The spontaneous rearrangement of residual atoms leads to the surface modification of the size, shape, and composition of the nanocrystals. For example, Pd/Pt core/shell nanodendrites were first synthesized via one‐step aqueous solution method with the assistance of block copolymers.^[^ ^94^ ^]^ Then, as‐synthesized Pd/Pt nanodendrites were selectively chemically etched under the presence of high‐concentrated nitric acid (**Figure** **10**a). After etching, the Pd core was partially dissolved, and the remaining Pd and Pt atoms were rearranged, inducing the formation of Pd/Pt dendritic nanocages (Figure 10b,c). Due to the abundant active sites on the interior and exterior surfaces, the MOR activity of this Pd/Pt dendritic nanocages was much higher than that of commercial Pt/C. Electrochemical dealloying is another method for inducing the atomic rearrangement by selectively removing the alloying metals with a lower reduction potential from the Pd‐based alloys via an electrochemical or a chemical approach, leading to create core/shell or nanoporous structures.^[^ ^48^ ^]^ Upon electrochemical cycling, Jana et al. transformed intermetallic PdCu_3_ nanoparticles synthesized from an oleylamine‐mediated strategy into dealloyed PdCu/Pd core/shell nanocatalysts.^[^ ^95^ ^]^ On account of the formation of low coordination number Pd active sites and the optimization of hydrogen adsorption, the *d*‐band center was altered, thus exhibiting the enhanced HER activity.

In terms of entropy, the phase segregation is often an unfavorable process for bimetallic nanocrystals because of the surface energy hard to change.^[^ ^96^ ^]^ However, increasing the thermal treatment temperature can affect the alloying behavior of two components in alloyed nanocrystals. Therefore, by annealing nanocrystals in the inert atmospheres, the internal noble metal atoms may be migrated to the surface or subsurface. Especially, the different atoms have strong difference in the enhancements of the diffusion rates in atmosphere annealing process. Using this principle, the surface‐segregated PdNi icosahedra were synthesized via one‐pot wet‐chemical method (Figure 10d).^[13c]^ The well‐designed PdNi icosahedra was composed of Pd icosahedra as the core and Ni as shell. When annealing under H_2_/Ar atmosphere, the annealing temperature controlled the degree of the migration. As the temperature increased to 400 °C, the surface structure of PdNi icosahedra was unchanged, while the PdNi icosahedra with Pd‐rich surface were obtained as the Pd atoms diffused outward (Figure 10e,f). Note that the optimized Pd_6_Ni icosahedra with surface Ni decoration show the highest ORR activity of 0.2 A mg^−1^_Pd_ (Figure 10g). The unique features of icosahedral Pd core and surface Ni segregation weaken the interaction between the Pd (111) facet and adsorbed oxygen, thus downshifting *d*‐band center for enhanced ORR activity. This can be confirmed by the fact that the ORR activity of surface Ni‐segregated Pd_6_Ni icosahedra was higher than those of conventional Pd_6_Ni icosahedra with alloy surfaces or Pd‐rich surfaces obtained from annealing Pd_6_Ni icosahedra with surface Ni decoration at different temperature (Figure 10h). Furthermore, this atmosphere annealing strategy was extended to the trimetallic PdCuRu nanocrystal systems.^[^ ^97^ ^]^ The surface Pd segregation of PdCuRu nanocrystals caused the significant enhancement of HER activity.

### 3.5 Interface Engineering

As a promising strategy for boosting performance of catalysts, the interfacial engineering, representing the interaction between multicomponent, brings a new opportunity for the enhanced catalytic activity, stability, and selectivity.^[^ ^98^ ^]^ Generally, the interface in catalysts refers to the boundary between different regions where electrocatalysis mainly occurs. The interfacial active sites can usually balance the adsorption/desorption for intermediates and tune the transportation of electron and mass, thus achieving the enhanced catalytic performance.^[^ ^99^ ^]^ As the development of interface engineering based on the advanced characterization technologies and theoretical calculations, an increasing number of Pd‐based electrocatalysts have been created. Summarizing the kinds of emerging interface engineered Pd‐based catalysts, the constructed interfacial nanostructures can be divided into metal–metal (alloy) interfaces, metal–compounds interfaces, and metal–supports interfaces. The construction methods toward interface engineering mainly include direct synthesis and posttreatment methods.

**Figure 11:**
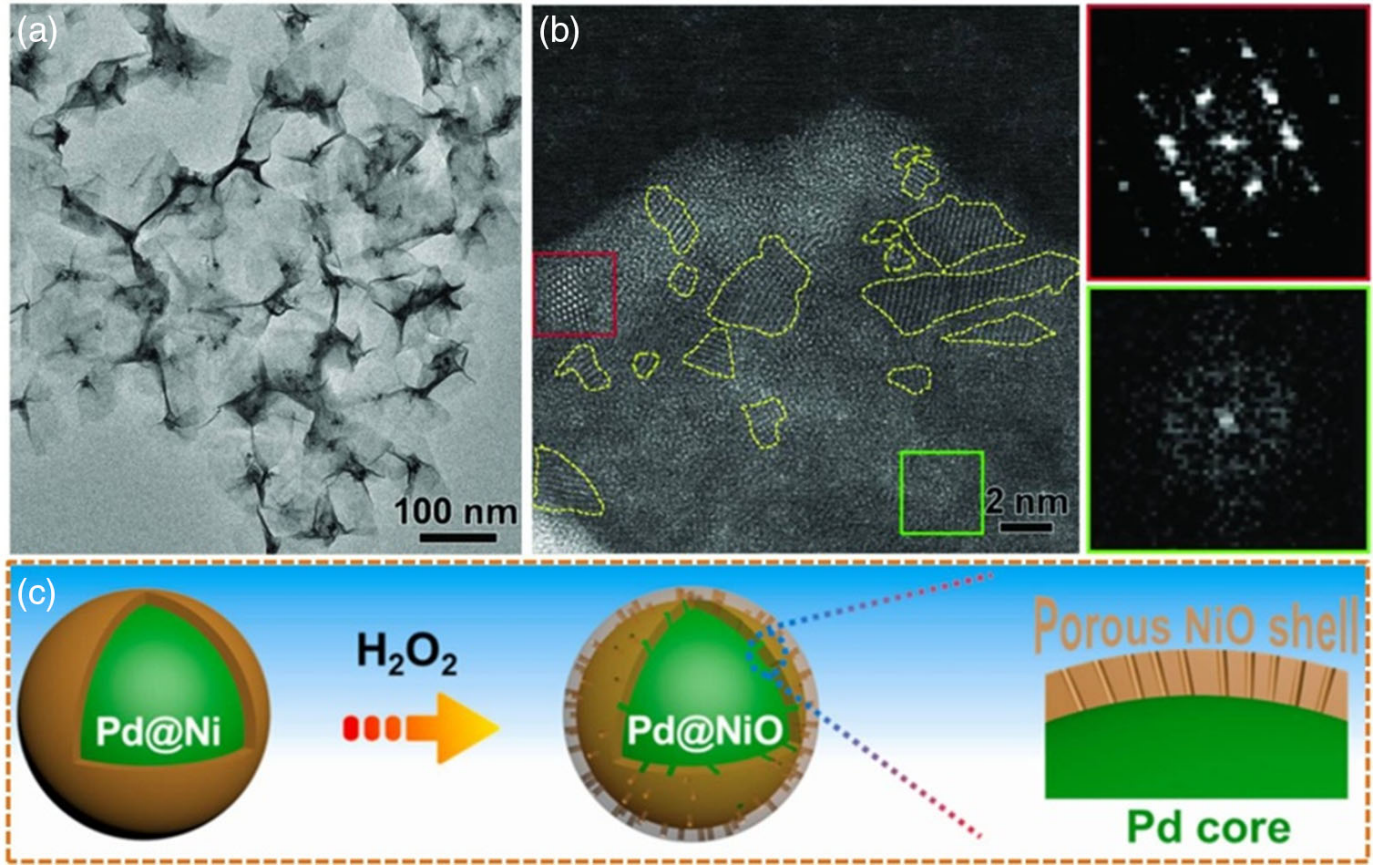
The typical strategy for enhancing electrocatalytic performance of Pd‐based nanoalloys by interface engineering. a) TEM image, b) aberration‐corrected HAADF‐STEM image of amorphous/crystalline hetero‐phase Pd nanosheets (the crystalline domains are marked by the dashed yellow curves, and the corresponding FFT patterns of the selected regions marked by the red and green squares, respectively). a,b) Reproduced with permission.^[^ ^100^ ^]^ Copyright 2018, Wiley‐VCH. c) Schematic illustration of the synthesis of Pd/NiO core/shell nanostructure. Reproduced with permission.^[13d]^ Copyright 2019, Elsevier.

The metal–metal (alloy) interfaces can be built in the alloyed nanocrystals, heterostructures, and core/shell structures. They show the attractive interests in optimizing electronic properties and atomic arrangement and stabilization. Yang et al. synthesized the amorphous/crystalline hetero‐phased Pd nanosheets with adjustable crystallinity via a simple wet‐chemical method (**Figure** **11**a,b).^[^ ^100^ ^]^ As the reaction temperature rises from 48 to 100 °C, the area of crystalline became stronger and stronger. The binding energy of stronger amorphous Pd nanosheets was greater, indicating the electronic structure had been changed on the amorphous/crystalline interfaces. Furthermore, the amorphous‐phase and crystalline‐phase‐dominant heterophased PdCu nanosheets were further constructed at a reaction temperature of 40 and 80 °C, respectively.^[^ ^101^ ^]^ The seed‐mediated growth induced the formation of core/shell and heterogeneous structures by using the colloidal seeds as growth templates. Using smooth Pd nanowires as seeds, spiny Pd/PtFe core/shell nanotubes were synthesized by growing Pt and Fe onto removable Pd nanowire substrates in Stranski–Krastanov mode with the galvanic dissolution of Pd cores.^[^ ^102^ ^]^ In addition, the Pd/PdAu/Pt core/shell/shell sandwiched nanowires were also fabricated by using Pd nanowires as seeds to deposit PdAu layers and Pt shell.^[^ ^103^ ^]^ These two interfacial nanowires (Pd/PtFe and Pd/PdAu/Pt) showed high ORR activity, attributed to the strong interaction between interlayers and shells.

The metal compounds have good stability for anchoring alloys, nanoclusters, and single atoms, thus providing strong interactions for boosting the catalytic activity and stability. The most widespread Pd(‐based)–compounds interface is metal–oxide interfaces, especially when the Pd atoms are easily reduced in an one‐pot process while the other atoms are not easily reduced, such as Pd/TiO_2_ system.^[^ ^104^ ^]^ Annealing Pd‐based materials or other special surface oxidation treatment can also be used to construct Pd—oxide interfaces. For example, Pd/NiO core/shell nanoparticles can be synthesized by annealing Pd/Ni core/shell nanoparticles in air atmosphere (Figure 11c).^[13d]^ By adjusting the surface chemical states of NiO, the interfacial *d*‐band‐offset was realized to overcome insurmountable high ORR barriers, making the Pd/Ni core/shell catalysts a kind of potential electrocatalysts for fuel cells and beyond. In addition to oxides, many other compounds, including metal sulfides, phosphides, hydroxides, and so on, have also been found to couple with Pd‐based materials for improving electrocatalytic performance.^[^ ^105^ ^]^ More recently, Bao et al. demonstrated that the formed Pd/FeP interfacial sites are more active than exclusive Pd sites.^[105d]^ By annealing inactivated Pd/FeP composites, Pd/FeP hereostructures with abundant interfacial sites were achieved, much more efficient for FAOR than pristine Pd/FeP. The mass activity of annealed Pd/FeP catalysts was 1.6 and 2.8 times higher than that of pristine Pd/FeP and commercial Pd/C. It is worth mentioning that building the interfaces between carbides/nitrides and Pd‐based materials are still a great challenge due to the high energy barrier required to generate the carbides and nitrides.

The low electrical conductivity of metal compounds presents a new challenge that the catalytically active sites can be covered due to the segregation of suboxide species derived from some specific reducible oxides. This issue can be addressed by choosing different high‐conductive support materials, such as ionic liquid, MXenes, N‐doped reduced graphene oxides, and carbon nanotubes.^[^ ^106^ ^]^ These substrates have a positive effect in electrocatalysis. Embedding Pd‐based alloys on N‐functionalized graphene has been confirmed to be a successful strategy for promoting electrocatalysis. Recently, multimetallic PdPtCu mesoporous hemispheres were embedded in N‐functionalized graphene to display high electrocatalytic activity and stability toward alcohol oxidation electrocatalysis.^[^ ^107^ ^]^ The synergistic effects between N‐functionalized graphene supports with high conductivity and PdPtCu hemispheres with low‐coordinate features accelerated the catalytic reaction kinetics.

## 4 Conclusions and Perspectives

In summary, designing Pd‐based electrocatalysts is presenting a feasible approach with attractive and exciting activities. Based on the theoretical and experimental experiences, we comprehensively recapped the recent advances in regulating the dimensionality of Pd based for enhanced electrocatalysis. To further boost the electrocatalytic performance of Pd‐based nanoalloys, the structural regulation is indispensable, including intermetallics, doping, defects, surface, and interface engineering. Benefitting from these advanced strategies, the adsorption/desorption behaviors can be optimized. In addition, the density and intrinsic activities of active sites are increased, thus achieving enhanced performance in several typical reactions. Despite the impressive progress have been obtained so far, there is still much room for the development of Pd‐based electrocatalysts with higher catalytic activity and more stable durability by regulating their electronic and orbital properties. To meet the future practical applications, we here supply several personal perspectives which may be considered in future attentions.

The first note to emphasize is the requirement to find a universal rule applicable to the structural regulation toward a specific reaction. It has been demonstrated that the different surface structure can significantly enhance electrocatalytic activities; however, different catalytic mechanisms are conducted in various reactions. If one could understand which properties of Pd catalysts are the key to enhancing the catalytic performance of specific reactions, one can obtain more effective methods to achieve Pd‐based electrocatalysts that are anticipated to break through the ceiling of activity.

In addition, we believe that the present strategies for structural regulation will continue to serve as powerful tools to boost the intrinsic activities of Pd‐based electrocatalysts. Nevertheless, the desired catalysts have not yet come out. Therefore, it is urgent to reveal the fine geometric and electronic structures of Pd‐based catalysts and construct structure–performance relationship. Moreover, creating novel Pd‐based nanostructures, combining with the advanced structural regulation, provides an expected possibility for boosting catalytic performance, such as single‐layer nanosheets that can provide maximum atom utilization.

Furthermore, it is also necessary to develop more advanced characterization techniques, such as in situ X‐ray absorption spectroscopy (XAS), XPS, Fourier transform infrared (FT‐IR) spectroscopy, vibrational sum‐frequency generation spectroscopy, and atomic‐resolution aberration‐corrected high‐resolution transmission electron microscopy, to be able to accurately detect reaction intermediates and clarify the active sites. Especially, the synergy between the various components should be clearly identified. The actual role of each active site should be also analyzed. Various in situ and ex situ characterizations are conducive to obtaining more precise structure and surface chemical states information, further stimulating the establishment of more accurate DFT calculative models to uncover the regulation essence for optimized structural properties.

Finally, to realize carbon neutrality, a novel environmental protection concept, electrocatalytic CO_2_RR, has attracted much attention, but a start‐up stage.^[^ ^108^ ^]^ Extending engineering structural properties into Pd‐based electrocatalysts is a feasible trend for enhanced performance toward CO_2_RR. In particular for selectivity, regulating the inherent catalytic behaviors to obtain value‐added C^2+^ products may be achieved on the Pd‐based nanomaterials by these interesting strategies.

Combining with the effects from chemistry, material science, and engineering, the advanced engineered structural features strategies will open new venues for the preparation of highly active and stable electrocatalysts in the future. We also anticipate that this general guidance can be of assistance in other designed electrocatalysts including other metal‐based and metal free‐based electrocatalysts.

## Conflict of Interest

The authors declare no conflict of interest.
